# A Preliminary Report of Network Electroencephalographic Measures in Primary Progressive Apraxia of Speech and Aphasia

**DOI:** 10.3390/brainsci12030378

**Published:** 2022-03-12

**Authors:** Rene L. Utianski, Hugo Botha, John N. Caviness, Gregory A. Worrell, Joseph R. Duffy, Heather M. Clark, Jennifer L. Whitwell, Keith A. Josephs

**Affiliations:** 1 Department of Neurology, Mayo Clinic, Rochester, MN 55905, USA; botha.hugo@mayo.edu (H.B.); worrell.gregory@mayo.edu (G.A.W.); jduffy@mayo.edu (J.R.D.); clark.heather1@mayo.edu (H.M.C.); josephs.keith@mayo.edu (K.A.J.); 2 Department of Neurology, Mayo Clinic, Scottsdale, AZ 85259, USA; jcaviness@mayo.edu; 3 Department of Radiology, Mayo Clinic, Rochester, MN 55905, USA; whitwell.jennifer@mayo.edu

**Keywords:** electroencephalography (EEG), network analysis, graph theory, primary progressive aphasia, progressive apraxia of speech

## Abstract

The objective of this study was to characterize network-level changes in nonfluent/agrammatic Primary Progressive Aphasia (agPPA) and Primary Progressive Apraxia of Speech (PPAOS) with graph theory (GT) measures derived from scalp electroencephalography (EEG) recordings. EEGs of 15 agPPA and 7 PPAOS patients were collected during relaxed wakefulness with eyes closed (21 electrodes, 10–20 positions, 256 Hz sampling rate, 1–200 Hz bandpass filter). Eight artifact-free, non-overlapping 1024-point epochs were selected. Via Brainwave software, GT weighted connectivity and minimum spanning tree (MST) measures were calculated for theta and upper and lower alpha frequency bands. Differences in GT and MST measures between agPPA and PPAOS were assessed with Wilcoxon rank-sum tests. Of greatest interest, Spearman correlations were computed between behavioral and network measures in all frequency bands across all patients. There were no statistically significant differences in GT or MST measures between agPPA and PPAOS. There were significant correlations between several network and behavioral variables. The correlations demonstrate a relationship between reduced global efficiency and clinical symptom severity (e.g., parkinsonism, AOS). This preliminary, exploratory study demonstrates potential for EEG GT measures to quantify network changes associated with degenerative speech–language disorders.

## 1. Introduction

### 1.1. EEG Graph Theory Measures

The use of electroencephalography (EEG) has expanded from identifying and characterizing seizure disorders to differentiating many different cerebral functions. Past research has demonstrated that clinical EEG is sensitive to dementia associated with Alzheimer’s (AD) [1] and Parkinson’s diseases (PD) [2], and nonfluent/agrammatic Primary Progressive Aphasia (agPPA) [3], but not Primary Progressive Apraxia of Speech (PPAOS; patients who present with isolated apraxia of speech (AOS)) [4]. However, clinical EEG studies describe overall brain health and do not quantify interactions among multiple brain areas, or network activity.

Graph theory is a branch of mathematics that is central to much of the modern “network neuroscience.” It is premised on representing a system or network as a collection of nodes, with the interaction among them represented by edges. Node, edge, subgraph, and global metrics can then be calculated and compared between groups or to a behavioral measure. For example, degree centrality is a node-level metric calculated as the number of edges, or the total weight of edges, to a given node. Nodes can be grouped in modules, representing nodes that tend to connect to each other more than other nodes and potentially reflect specialized processing. Some high-degree nodes connect many modules and are referred to as hubs. At a global scale, most real-world networks balance integration, or a high level of connectivity between nodes, and segregation, reflecting distinct modules in a network. The extent to which this balance is optimized is captured in the small world-ness of the network. In EEG studies, the nodes are represented by the electrodes and the edges by a measure of coherence within a selected frequency band [5].

### 1.2. EEG Graph Theory in Neurodegenerative Disease

Studies have shown changes in EEG graph theory measures in dementia associated with PD [6], AD [7,8,9,10], and frontotemporal dementia (FTD) [11]. More specifically, EEGs of cognitively unimpaired patients with PD showed increased local integration across frequency bands when compared to cognitively unimpaired controls; those with dementia associated with PD had decreased integration in the lower alpha band relative to the cognitively unimpaired PD patients [6], suggesting the latter change was related to cognitive changes, not simply the presence of the disease. Analysis of brain networks of patients with AD-related dementia have shown decreased connectivity (or increased randomness), with loss of hubs compared to cognitively unimpaired controls [9,10].

Different types of network change have been shown in FTD. There were no differences in clustering coefficient or path length measures; however, the lower alpha band degree correlation increased in FTD relative to cognitively unimpaired controls, suggesting reduced segregation [11]. Overall, while AD patients showed less order, FTD patients showed a more ordered structure, possibly reflecting the differing underlying pathophysiology. However, in that study, the behavioral variant and semantic dementia were the only clinical phenotypes represented. Overall, it seems that patterns of network breakdown may be evident in neurodegenerative cognitive disorders and may be specific to the clinical syndromes and/ or causative pathology. To date, EEG graph theory measures have not been described in PPAOS and only one study has addressed this in agPPA [12], two other clinical syndromes associated with FTD pathology.

### 1.3. Primary Progressive Aphasia and Apraxia of Speech

Briefly, PPA encompasses a group of neurodegenerative syndromes characterized by progressive and predominant language impairment [13]. The agPPA subtype is characterized by grammatical errors in speech and writing and, not infrequently, accompanied by AOS, a motor speech disorder characterized by disruption in sensorimotor planning and/or programming [14]. When AOS, and not aphasia, is the initial manifestation of neurodegenerative disorders it is referred to as PPAOS [15,16]. In the context of PPAOS, some patients eventually develop aphasia that remains milder in severity than the AOS [17]. Research has suggested the initial or combination of speech (i.e., AOS) and language (i.e., aphasia) features may have implications for imaging findings, underlying pathology and the anticipated progression of the neurodegenerative disorder [18,19,20,21]. Given that more cortical imaging findings have been associated with the presence of aphasia, we opted to group those with aphasia, with or without AOS and regardless of predominance, into a single group referred to as agPPA. Many patients with PPAOS have normal MRIs, with FDG-PET considered the most sensitive imaging biomarker [22]. Unfortunately, FDG PET scans are not ubiquitously available and are sometimes cost-prohibited. 

### 1.4. Present Study

The primary goal of this study was to provide foundational information on which to build our understanding of the network breakdowns in patients with progressive AOS and/or aphasia. Ultimately, this might inform our theoretical understanding of the neuropathophysiology underlying these clinical presentations, and clinically, inform a more widely available and cost-effective method to support differential diagnosis. Toward that end, we describe graph theory network measures and correlate them with indices of speech and language deficits to better understand their relationship.

## 2. Materials and Methods

### 2.1. Participants

The study was approved by Mayo Clinic’s Institutional Review Board (#17-002468 on 19 July 2017); all patients were native English speakers and gave written consent according to the Declaration of Helsinki. Between October 2016 and December 2019, a total of 22 patients with agPPA (*n* = 15) or PPAOS (*n* = 7) completed a clinical EEG recording as part of a larger study conducted by the Neurodegenerative Research Group (NRG).

### 2.2. Clinical Measures

A comprehensive speech–language evaluation was conducted by an experienced speech–language pathologist (SLP). Clinical judgments regarding the presence, nature (i.e., type), and severity of AOS and aphasia were made by the examining clinician and subsequently confirmed by consensus agreement with at least one other non-examining SLP. The SLPs were experienced in differential diagnosis of neurodegenerative speech and language disorders.

Severity ratings reflected gestalt clinical judgment on a 5-point scale (0 = absent, 1 = mild, 2 = moderate, 3 = marked, 4 = severe). Other formal measures were administered and used to inform the overall judgments. The Western Aphasia Battery-Revised (WAB-R) Aphasia Quotient (WAB-AQ) [23], as a composite measure of global language ability, and the Northwestern Anagram Test (NAT) [24], a non-speech sentence-production task, were administered. A conversational speech sample, including narrative picture description, was collected as a part of the WAB-R. Additionally, supplementary speech and speech-like tasks (alternating and sequential motion rates) were elicited. The speech samples were used to reach consensus about the predominance of phonetic or prosodic speech characteristics by the same SLPs, as previously described [25]. The speech samples were also used to score the Apraxia of Speech Rating Scale—version 3 (ASRS-3) [25,26], an index of abnormal speech features and severity of AOS.

As part of the neurological evaluation, the Montreal Cognitive Assessment (MoCA) [27], a screening test of general cognition, was completed. The Movement Disorder Society-Sponsored Revision of the Unified Parkinson’s Disease Rating Scale, Motor section (MDS-UPDRS III) [28], an index of motor functioning, was scored.

### 2.3. Electroencephalographic (EEG) Recording

Scalp EEG recordings were collected with XLTEK utilizing 21 electrodes placed with standard 10–20 positions, recording reference electrode of CPZ, a sampling rate of 256 Hz, 1 Hz low-frequency filter, and 70 Hz high-frequency filter, during relaxed wakefulness, wherein patients sat quietly with their eyes closed for 90% of the 45 to 55 min recording. A Natus EMU40EX Wireless LTM Amplifier (Natus Medical Incorporated, CA, USA) was utilized. A time base of 30 mm/sec with patient-individualized sensitivity was utilized for ongoing monitoring of artifacts. Clinical protocols for “awake” EEG were followed; no request was made for sleep deprivation. Recording intervals that included mental activation were not included for analysis.

### 2.4. EEG Processing

The continuous EEG data were divided into non-overlapping 1024-point (1023 ms) epochs, dictated by the sampling rate (256 Hz). Each epoch was visually inspected for artifacts, though rejection of artifacts was uncommon due to the vigilant monitoring of the online acquisition. For detecting blinking and other eye-movement artifacts, comparison was made to the vertical and horizontal eye movement channels. Epochs with muscle artifacts were rejected if such artifact signals were present grossly. No specific criteria were applied, but rather gestalt judgment. Consistent with prior research [6], 8 artifact-free epochs were chosen for analysis. 

### 2.5. Graph Theory Analysis

Graph theory network analysis was performed with Brainwave software (http://home.kpn.nl/stam7883/brainwave.html, accessed on 27 January 2022). Briefly, functional connectivity was assessed with phase lag index (PLI), as research has shown it is less affected by volume conduction than other measures [29]. Complementary traditional graph theory weighted connectivity and minimum spanning tree (MST) measures [30,31] were selected. Selected measures are shown in Table 1. All graph theory and MST measures were calculated for the following frequency bands (Hz): theta (4–8), alpha1 (8–10), and alpha2 (10–13), selected given prior demonstration of slowing and alterations in these ranges [4].

Utilizing Brainwave, the weighted network map of connections and minimum spanning tree were visualized for a given frequency band; this was performed for the whole cohort and separately for each subgroup (PPAOS and agPPA) based on an average of all individual epochs. In the weighed network map, the lines represent connections with PLI synchronization above the noted connectivity threshold.

### 2.6. Statistical Analysis

Differences in clinical characteristics between subgroups were assessed with Wilcoxon rank-sum tests. Differences between agPPA and PPAOS patients’ graph theory and MST measures were assessed separately with Wilcoxon rank-sum tests, each collapsed across frequency bands. Spearman correlations were computed between behavioral and network measures in all frequency bands across all patients. Statistical analyses were performed utilizing the JMP computer software (JMP Software, version Pro 14; SAS Institute Inc., Cary, NC, USA) with significance set at *p* < 0.05. Multiple comparison corrections were not imposed due to the small sample size. Given the exploratory nature of the study, we prioritized avoiding type II error inflation which unfortunately results from all common multiple comparison corrections (see Figure 5 in the following reference) [32].

## 3. Results

Demographic information and clinical data for the cohort and each subgroup are detailed in Table 2. Overall, agPPA patients were slightly younger, with slightly longer disease durations, compared to PPAOS patients. Sex representation was equivalent (approximately 60% female in each group). Consistent with the diagnoses, indices of language functioning (e.g., NAT and WAB-AQ) were lower in agPPA compared to PPAOS. Scores on the index of general cognition (the MoCA) were lower and ratings of parkinsonism (on the MDS-UPDRS III) were slightly higher in agPPA compared to PPAOS. There was no difference in AOS severity or ASRS-3, a quantitative index of AOS, between subgroups. For all patients, objective testing aligned with the SLP’s gestalt clinical judgment (i.e., normal language testing for those diagnosed PPAOS).

**Figure 1:**
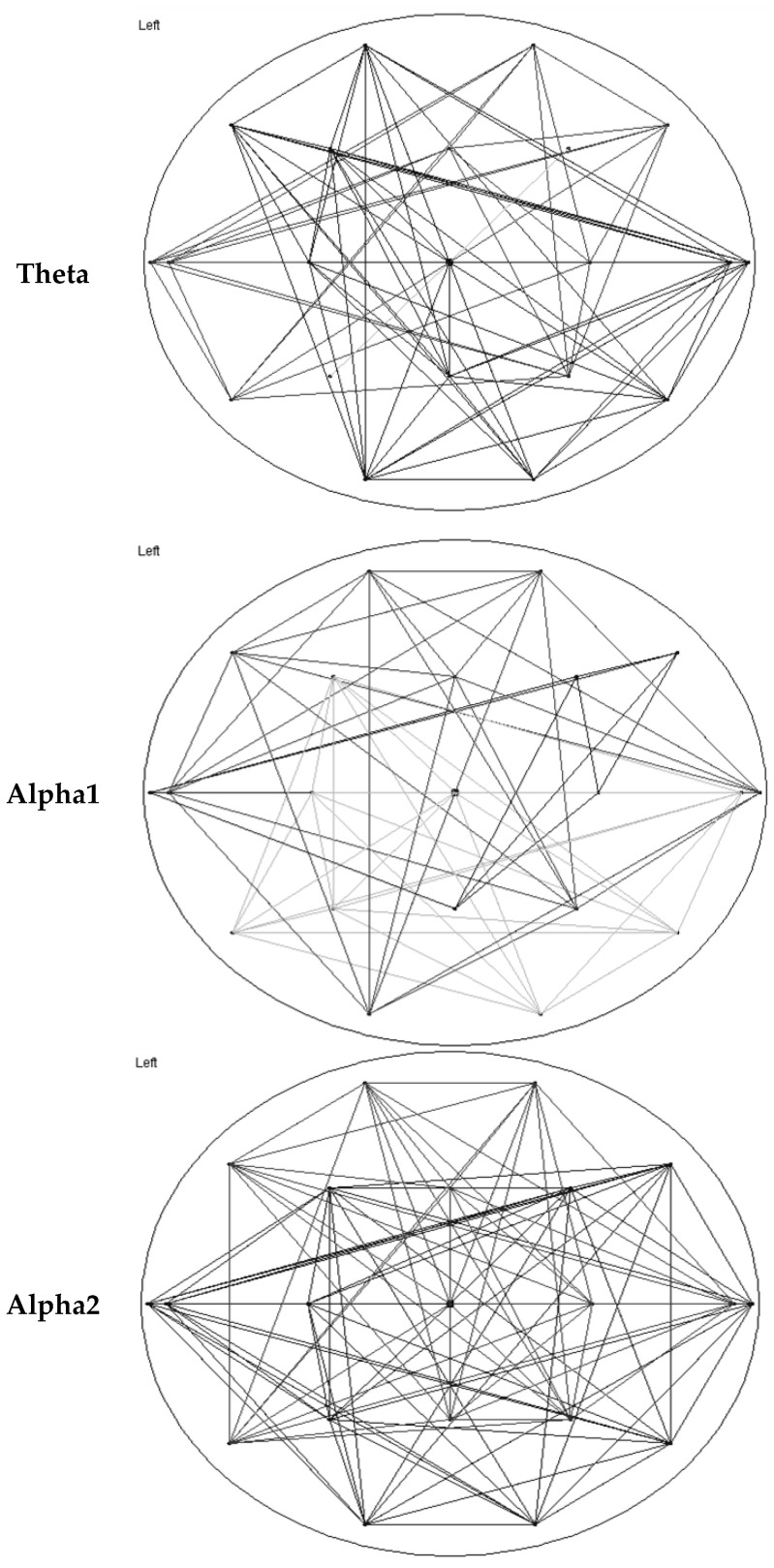
Average network maps for all patients for the theta, alpha1, and alpha2 frequency bands. The maps demonstrate the presence of correlations between pairs of channels, with threshold of 0.1 (PLI value) or correlations above that threshold.

**Figure 2:**
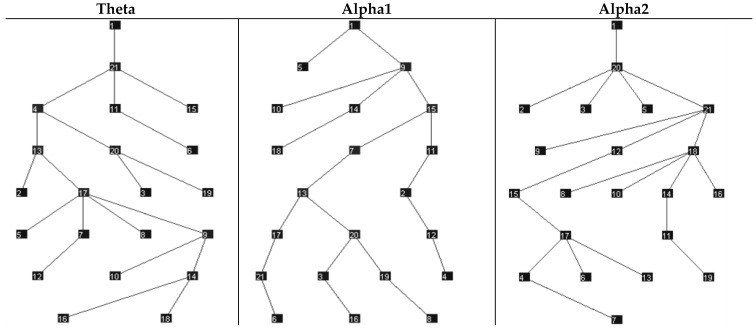
Minimum Spanning Trees for the cohort of all patients for the theta, alpha1, and alpha2 frequency bands. This visualization connects all nodes, maximizing synchronization. The numbers reflect electrode numbers; consistent with assessing mean connectivity, the relationships between specific electrodes were not explored in this study.

**Figure 3:**
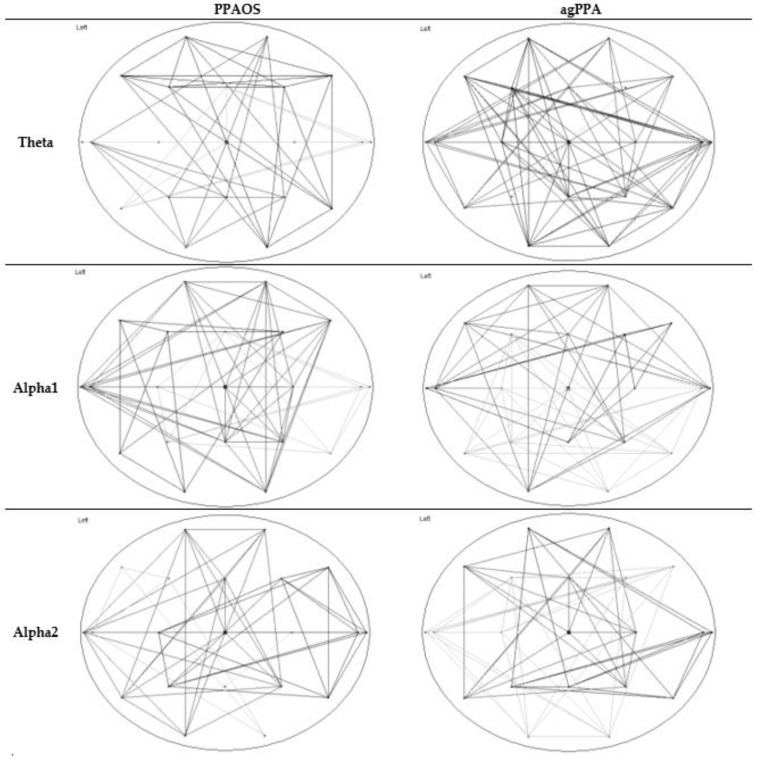
Average network maps separating the agPPA patients and PPAOS, for the theta, alpha1, and alpha2 frequency bands. The maps demonstrate the presence of correlations between pairs of channels, with threshold of 0.1 (PLI value) or correlations above that threshold.

**Figure 4:**
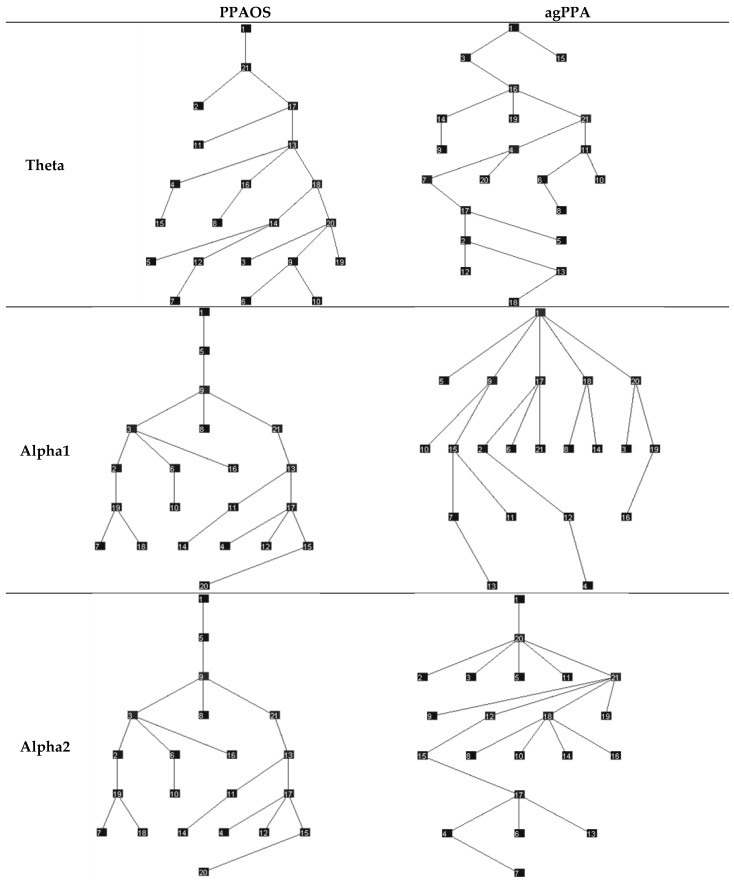
Minimum Spanning Trees (MSTs) separating the agPPA patients and PPAOS, for the theta, alpha1, and alpha2 frequency bands. This visualization connects all nodes, maximizing synchronization. The numbers reflect electrode numbers; consistent with assessing mean connectivity, the relationships between specific electrodes were not explored in this study.

Median network measures are reported in Table 3. Omnibus tests of differences did not support significant differences in either graph theory or MST measures between agPPA and PPAOS. The data are visualized in power maps and minimum spanning trees; results for the whole cohort are presented in Figure 1 and Figure 2, respectively. Data were additionally visualized relative to the subgroups of agPPA and PPAOS, shown in Figure 3 and Figure 4. The power maps show differences in the distribution of connectivity for agPPA compared to PPAOS. The MSTs for the agPPA in the alpha frequency bands show a relatively more “star-like” quality, with a more central node connecting to the majority of other nodes. The star-like quality typically relates to a more integrated network, with a smaller diameter and shorter path length; this MST configuration typically reflects efficient information transfer, although not always. One possible downfall is information overload at the central node with subsequent inefficiency. 

To better understand the relationship between graph theory measures and clinical presentations, non-parametric correlations between network and behavioral variables were calculated across all patients; these are reported in Table 4. Statistically significant relationships were identified between: age and alpha2 gamma (ρ = −0.60), kappa_w_ (ρ = −0.42), and MST leaf (ρ = −0.48); disease duration and theta modularity (ρ = −0.58); disease duration and alpha1 lambda (ρ = 0.78); MDS-UPDRS III and alpha1 PLI (ρ = −0.55) and kappa_w_ (ρ = −0.56); MDS-UPDRS III and alpha2 MST leaf (ρ = −0.47); ASRS-3 and alpha1 gamma (ρ = 0.54) and lambda (ρ = 0.82); ASRS-3 and alpha2 lambda (ρ = 0.059). No significant relationships identified between graph theory or MST measures and the MoCA or WAB-AQ. Correlation scatter plots, with individual data points indicating group membership, are provided in Supplementary Materials.

## 4. Discussion

### 4.1. General Discussion

The results provide EEG evidence of network alteration in patients with agPPA and PPAOS. While it is difficult to fully describe or dismiss significant differences between groups due to the sample sizes, this exploratory study demonstrates potential for EEG graph theory measures to quantify network changes associated with degenerative speech and language disorders. The novelty of this study is the patient population and the correlation between EEG graph theory measures and certain clinical measures.

The results broadly suggest that increased global integration, or reduced network specificity, occurs in degenerative speech and language disorders. These network changes exist even in the absence of strong evidence for structural changes on magnetic resonance imaging [4] and it is therefore considered unlikely these are artifacts of atrophy. The visualization of the data supports the presence of network alterations, with correlation analyses offering insight into their clinical manifestations. This study explored global connectivity, rather than that of smaller cortical regions, which should be the focus of future studies. Further, it is not yet clear if the network changes represent direct disease effects or a compensatory response. For example, additional regional graph theory measures and correlational analyses might clarify whether connectivity in the region of suspected disease (e.g., precentral gyrus or supplementary motor area) is reduced and/ or whether there are downstream effects of hyperconnectivity in other areas working to compensate for that loss; alternatively, if hyperconnectivity is seen in the region of disease, it might reflect system stress. A more complete understanding of network disruption in neurodegenerative speech and language disorders, perhaps in the context of the cascading network failure model [33], might better elucidate the relationship between the underlying pathophysiology and clinical presentation. Toward that end, future studies will explore the graph theory measures and relationships with clinical measures longitudinally.

### 4.2. Tests of Differences and Correlations

In this study, the agPPA patients were, on average, slightly younger with slightly longer disease durations compared to PPAOS patients. These differences warrant caution when comparing the graph theory measures between the two groups. Scores on the index of general cognition (the MoCA) were lower and ratings of parkinsonism (on the MDS-UPDRS III) were also slightly higher in agPPA compared to PPAOS. However, it is important that there was no difference in AOS severity or ASRS-3, a quantitative index of AOS, between the subgroups. 

Tests of differences did not support significant differences in either graph theory or MST measures between agPPA and PPAOS patients. Interestingly, differences in clinical EEGs were seen between the groups (i.e., relative to the presence of aphasia) [4] in a smaller subset of those patients included in this study, which is more consistent with the visualization of the data. In that study, patients with PPAOS (*n* = 5) had normal EEGS while two of three those with aphasia had theta slowing. The power maps and minimum spanning trees for the whole cohort (Figure 1 and Figure 2, respectively) do not equally reflect the visualization of the agPPA and PPAOS subgroups (Figure 3 and Figure 4). The MSTs for the agPPA in the alpha1 and alpha2 frequency bands show a more “star-like” quality, although given the unequal sample sizes, this should be interpreted cautiously. Future studies should systematically explore other possible sources of differences, including the subtype of AOS (i.e., phonetic or prosodic predominant speech disturbance [27]).

The correlation analysis offers insight into the relationship between graph theory measures and clinical presentations (see Table 4) and provides complementary support for reduced global efficiency and increased integration in patients with agPPA and PPAOS. There were negative relationships between the MDS-UPDRS III, a measure of motor impairment, and synchronicity, kappa, and MST leaf in the alpha band, likely reflecting severity (reduced synchronicity with increased motor dysfunction). The strongest correlation was noted between the ASRS-3, a measure of AOS severity, and lambda in the alpha1 frequency band, suggesting a relationship between reduced distance between nodes (measured by lambda) and more prominent AOS (indexed by the ASRS). This relationship supports the notion that network measures may better reflect more abstract process breakdowns (such as that of sensorimotor planning/programming in AOS) that have a less clear structural correlate, particularly compared to clinical EEG reads which were reportedly normal in these patients [4]. Interestingly, there were no significant relationships identified between graph theory or MST measures and the MoCA or WAB-AQ. This work lays the foundation to better understand whether these relationships (and lack thereof) represent the loss of ordered correlations (or anti-correlations) resulting from the disease. Frequency band differences require further exploration.

### 4.3. Relationship with Functional Connectivity Literature

While this is the first study of EEG graph theory measures in PPAOS, the broader literature on neurodegenerative disease provides helpful context for these findings. A recent study showed promising utility of EEG graph theory measures, in conjunction with machine learning, in distinguishing patients with PPA from controls [12]; however, the focus of the study was the machine-learning algorithms rather than the graph theory measures themselves. EEGs from patients with dementia associated with Lewy bodies had reduced connectivity strength in the alpha frequency band relative to cognitively unimpaired controls and patients with dementia from Alzheimer’s disease, with additional evidence of reduced network efficiency. There were associations with clinical measures, including between leaf fraction and the Mini-Mental State Examination, a test of general cognition [34]. Another study showed increased connectivity in the theta band in patients with Alzheimer’s disease dementia and mild cognitive impairment, relative to cognitively unimpaired controls; the connectivity measures were also correlated with neuropsychological test scores [35]. Finally, assessment of functional connectivity in multiple sclerosis via magnetoencephalography showed a less integrated network related to more severe cognitive impairment [36]. Together, these and other recent studies support the practical implications of EEG graph theory for accurate diagnosis, early detection, and disease monitoring [37]. It may be that a relative combination of graph theory metrics and their clinical correlates are most sensitive for diagnostic precision.

While a different modality, there have been at least four studies of functional connectivity in PPA and PPAOS via fMRI [38,39,40,41]. These studies have broadly demonstrated reduced connectivity in these populations. An fMRI study of functional connectivity in patients with PPAOS demonstrated reduced connectivity, specifically in the supplementary motor areas (SMA); reduced connectivity in the right SMA negatively correlated another measure of AOS, an articulatory error score, while connectivity in the left working memory network correlated with the ASRS [38]. These can serve as a foundation from which to formulate hypotheses for future regional analyses; for instance, it is hypothesized that there may be loss of ordered synchronization between frontal regions, supplementary motor areas, and, overall, regions in the left hemisphere compared to others. 

Other fMRI studies of agPPA patients [40], patients with semantic variant PPA [39], and PPA patients more broadly [41] showed lower global integration and alteration in hub distribution in speech-predominant regions compared to cognitively unimpaired controls that were not entirely explained by structural changes. Taken together, there is support for looking at more functional, rather than structural, measures of disease burden in understanding clinical symptoms. 

### 4.4. Limitations and Future Directions

There are limitations to the current study. While this is the largest documented EEG study of patients with PPAOS, the sample size was relatively small, which limited our ability to examine smaller subgroup influences (e.g., AOS type, phonetic or prosodic) on the findings. Further, given the results of the power analysis (which suggests the need for a much larger sample size; details not reported for brevity), we are unable to assess robust effects from this sample size. We are lacking an ideally age- and sex-matched cognitively unimpaired control cohort to expand the impact beyond patient group description and to assess the diagnostic power between impaired and unimpaired groups. The patient group comparisons offer important insight on which to base future hypotheses, but the groups are imbalanced in size, age, and disease duration. To explore the complex relationship between EEG network measures, clinical symptoms, and other explanatory variables (such as age and disease duration), regression models should be considered with relevant covariates.

The novelty of the current study lies in the relationship of network measures and clinical parameters. Stronger relationships are expected between regional, rather than mean, network measures, which should be explored in future studies. Additional limitations are methodological, including the use of 21 electrodes and a 256 Hz sampling rate, as well as PLI in favor of synchronization likelihood, another connectivity measure; different parameters, including exploring frequency bands beyond alpha and theta and frequency band measure ratios, could yield different results. Another modifiable parameter is sample length; here, the epoch length was limited by the sampling rate. While “clean” epochs were selected, no specific criteria were applied, which could impact replicability. Finally, differences in number of epochs and use of other connectivity measures could have influenced results [42], as could have the reference electrode [43]. While the recording parameters make it difficult to compare the results to those of published controls or other patient populations, methodological decisions were made to expedite transfer of these findings to clinical practice, which is considered a relative strength. Longitudinal assessments in a larger cohort, across the clinical severity spectrum and with different clinical phenotypes will also strengthen the interpretability and utility of these findings.

## 5. Conclusions

This study provides EEG evidence of network alteration and breakdown associated with primary progressive aphasia and apraxia of speech, although quantifiable differences between the groups are not yet clear. Nonetheless, this study demonstrates potential for EEG graph theory measures to quantify network changes that may reflect degenerative speech and language disturbances, given correlations with clinical measures. It remains important to compare these patterns to a healthy cognitively unimpaired control group. Describing network pathophysiology may have utility for understanding these diseases in a way not previously available, and, importantly, via a widely available and cost-effective method. This method may parlay into diagnostic EEG biomarkers, and ultimately, biomarkers for predicting disease progression and monitoring treatment-mediated improvements.

## Figures and Tables

**Table 1:** Definition of utilized network measures (adapted from Van Steen [5]).

Measure	Definition
PLI, Phase lag index	Measure of functional connectivity between nodes
Gamma, Normalized weighted clustering coefficient	Measure of connectivity between nodes or the extent to which neighboring nodes are also neighbors with one another, calculated per node and averaged over the entire network.
Lambda, Normalized characteristic path length	Measure of the average number of connections in the shortest path between two nodes of the network
Kappa_W,_ Weighted degree divergence	Measure of the broadness of the weighted degree distribution, where weighted degree is the summed weights of all edges connected to a node
Modularity	Measure of the degree to which nodes are more connected to each other than to nodes outside a given cluster (i.e., module)
MST BC_max,_ Maximum MST betweenness centrality	Maximum number of paths between any two MST nodes running through a single node
MST Diameter	Maximum number of connections (distance) between two MST nodes
MST Eccentricity	Average maximum distance between any two MST nodes
MST Leaf, MST leaf fraction	Measure of the number of MST nodes with only one link relative to the maximum possible number of leaves

**Table 2:** Median clinical and demographic information for this cohort and subgroups.

	agPPA (*n* = 15)	PPAOS (*n* = 7)	All (*n* = 22)
Age at EEG *	69	74	73
Disease Duration at EEG *	4.1	2	3.95
Sex	9 F (60%)	4 F (57%)	13 F (59%)
MoCA* (/30)	21	27	25
MDS-UPDRS III (/81) *	15	12	15
ASRS-3 (/52)	21	16	21
NAT (/10) *	5	9	7
WAB-AQ (/100) *	88.775	97.9	96.4
Aphasia Severity (/4) *	1.5	0	1
AOS Severity (/4)	2	2	2

Note: Age and disease duration (years); MoCA = Montreal Cognitive Assessment; MDS-UPDRS III = Movement Disorder Society-sponsored version of the Unified Parkinson’s Disease Rating Scale, Motor section; ASRS-3 = Apraxia of Speech Rating Scale-3; NAT = Northwestern Anagram Test; WAB-AQ = Western Aphasia Battery Revised Aphasia Quotient. Maximum score noted in row header, when applicable. Asterisk in row header indicates significant non-parametric test of differences between agPPA and PPAOS groups (*p* < 0.05).

**Table 3:** Median (interquartile range) for group-level network measures.

Measure	agPPA (*n* = 15)	PPAOS (*n* = 7)	All (*n* = 22)
***Theta***			
PLI	0.189 (0.180, 0.211)	0.187 (0.175, 0.209)	0.188 (0.178, 0.210)
Gamma	1.020 (1.007, 1.038)	1.030 (1.008, 1.040)	1.025 (1.007, 1.039)
Lambda	0.934 (0.932, 0.945)	0.934 (0.922, 0.947)	0.934 (0.930, 0.946)
Kappa_W_	4.001 (3.787, 4.439)	3.978 (3.694, 4.440)	3.998 (3.723, 4.439)
Modularity	0.077 (0.068, 0.083)	0.081 (0.070, 0.082)	0.078 (0.070, 0.082)
MST BC_max_	0.723 (0.692, 0.742)	0.711 (0.680, 0.722)	0.711 (0.691, 0.734)
MST Diameter	0.425 (0.413, 0.444)	0.406 (0.394, 0.438)	0.422 (0.405, 0.439)
MST Eccentricity	0.340 (0.324, 0.352)	0.330 (0.313, 0.347)	0.339 (0.323, 0.348)
MST Leaf	0.550 (0.519, 0.569)	0.544 (0.531, 0.575)	0.547 (0.530, 0.570)
***Alpha1***			
PLI	0.242 (0.242, 0.272)	0.257 (0.234, 0.274)	0.248 (0.234, 0.273)
Gamma	1.029 (1.022, 1.043)	1.033 (1.022, 1.039)	1.030 (1.022, 1.040)
Lambda	0.938 (0.937, 0.946)	0.935 (0.932, 0.941)	0.938 (0.935, 0.946)
Kappa_W_	5.143 (4.992, 5.753)	5.434 (4.940, 5.833)	5.296 (4.980, 5.773)
Modularity	0.079 (0.073, 0.084)	0.081 (0.070, 0.084)	0.080 (0.072, 0.084)
MST BC_max_	0.721 (0.707, 0.734)	0.733 (0.696, 0.757)	0.723 (0.705, 0.739)
MST Diameter	0.431 (0.388, 0.444)	0.394 (0.388, 0.431)	0.419 (0.388, 0.444)
MST Eccentricity	0.339 (0.306, 0.350)	0.314 (0.310, 0.348)	0.335 (0.309, 0.349)
MST Leaf	0.550 (0.531, 0.588)	0.581 (0.531, 0.600)	0.553 (0.531, 0.595)
***Alpha2***			
PLI	0.215 (0.193, 0.241)	0.207 (0.198, 0.219)	0.210 (0.196, 0.230)
Gamma	1.041 (1.012, 1.057)	1.029 (1.005, 1.044)	1.033 (1.012, 1.046)
Lambda	0.943 (0.932, 0.950)	0.933 (0.925, 0.938)	0.936 (0.928, 0.946)
Kappa_W_	4.553 (4.054, 5.157)	4.327 (4.242, 4.620)	4.440 (4.156, 4.920)
Modularity	0.075 (0.071, 0.086)	0.080 (0.071, 0.080)	0.077 (0.071, 0.084)
MST BC_max_	0.714 (0.700, 0.749)	0.734 (0.684, 0.742)	0.719 (0.700, 0.742)
MST Diameter	0.419 (0.388, 0.438)	0.406 (0.400, 0.419)	0.413 (0.398, 0.433)
MST Eccentricity	0.336 (0.309, 0.347)	0.320 (0.314, 0.332)	0.325 (0.314, 0.341)
MST Leaf	0.556 (0.538, 0.594)	0.563 (0.519, 0.581)	0.559 (0.536, 0.583)

**Table 4:** Non-parametric Spearman correlations between graph theory network and behavioral variables of interest.

	Age	Disease Duration	MoCA	MDS-UPDRS III	ASRS-3	WAB-AQ
***Theta***						
PLI	−0.1404	0.2893	−0.1254	0.0023	0.0788	−0.0589
Gamma	0.0583	0.1509	0.1904	−0.0736	0.0037	0.1218
Lambda	−0.1130	0.1524	−0.0325	−0.1586	−0.0283	−0.0558
Kappa_W_	−0.1512	0.2995	−0.1005	−0.0068	0.0640	−0.0392
Modularity	−0.0261	−0.5790 *	0.1266	−0.0739	−0.0382	0.1539
MST BC_max_	−0.0798	0.0590	−0.2501	0.1687	−0.1280	−0.3221
MST Diameter	0.2293	0.2937	−0.0906	0.0923	0.3820	0.0083
MST Eccentricity	0. 3000	0.2640	−0.0495	0.0977	0.3879	−0.0021
MST Leaf	−0.3106	−0.0500	0.1542	−0.1328	−0.0315	0.0990
***Alpha1***						
PLI	−0.3447	−0.0483	0.1778	−0.5537 *	−0.2069	0.1552
Gamma	0.0476	0.1421	0.1538	−0.1954	0.5357 *	0.2598
Lambda	0.0340	0.7833 *	−0.2614	0.4002	0.8246 *	−0.0485
Kappa_W_	−0.3505	−0.0596	0.1466	−0.5593 *	−0.1625	0.1869
Modularity	0.0986	0.0301	0.2071	0.2310	0.0197	0.1260
MST BC_max_	−0.0541	−0.2585	0.1364	−0.2593	−0.1016	0.0015
MST Diameter	0.0390	0.0542	−0.2057	0.2736	0.2427	0.0156
MST Eccentricity	−0.0456	0.1078	−0.1194	0.2591	0.1932	0.1147
MST Leaf	−0.0011	0.1291	0.1326	−0.3163	−0.0167	−0.0109
***Alpha2***						
PLI	−0.3771	0.0556	−0.1609	−0.0668	0.1822	0.0743
Gamma	−0.5991 *	0.0562	0.0650	−0.4110	0.1994	0.3263
Lambda	0.0102	0.3142	−0.2803	0.0221	0.5871 *	−0.1249
Kappa_W_	−0.4241 *	0.0153	−0.0688	−0.1545	0.1883	0.1735
Modularity	0.2749	−0.1153	0.1483	0.1116	−0.4380	−0.1314
MST BC_max_	−0.2936	0.3081	−0.0643	−0.0856	0.4436	0.3390
MST Diameter	0.3636	−0.0558	−0.0065	0.1252	−0.1891	−0.3696
MST Eccentricity	0.3539	−0.1016	−0.0295	0.1432	−0.2905	−0.4027
MST Leaf	−0.4824 *	0.0546	0.0546	−0.4686 *	0.0722	0.2962

Note: Age and disease duration (years); MoCA = Montreal Cognitive Assessment; MDS-UPDRS III = Movement Disorder Society-sponsored version of the Unified Parkinson’s Disease Rating Scale, Motor section; ASRS-3 = Apraxia of Speech Rating Scale-3; WAB-AQ = Western Aphasia Battery Revised Aphasia Quotient. Significant correlations (*p* < 0.05) are indicated by bold font and *; correction for multiple comparisons was not applied.

## Data Availability

Requests for the data presented in this study should be sent to the corresponding author for consideration.

## Supplementary Materials

The following supporting information can be downloaded at https://www.mdpi.com/article/10.3390/brainsci12030378/s1. Figure S1: Correlation scatter plots between graph theory and behavioral measures, with individual data points indicating frequency band and group membership.
