# Supplementary material for: A Preliminary Report of Network Electroencephalographic Measures in Primary Progressive Apraxia of Speech and Aphasia
Source: Brain Sci. 2022 Mar 12;12(3):378. doi: 10.3390/brainsci12030378 (PMC8946002; doi:10.3390/brainsci12030378)
Supplement: Supplementary file 1 [file brainsci-12-00378-s001.zip › brainsci-1594846-supplementary.pdf]

Supplementary Data.

Figure S1: *Correlation scatter plots between graph theory and behavioral measures, with individual data points indicating frequency band and group membership.*

Note: Duration = disease duration (from symptom onset; years); Age at exam (years); MoCA = score of Montreal Cognitive Assessment (maximum score 30); UPDRS-III = Movement Disorder Society-Unified Parkinson's Disease Rating Scale III (Motor Examination, maximum score 72); ASRS-3 = Apraxia of Speech Rating Scale- version 3 (maximum score 52); WAB- AQ Western Aphasia Battery (Revised) Aphasia Quotient (maximum score 100).

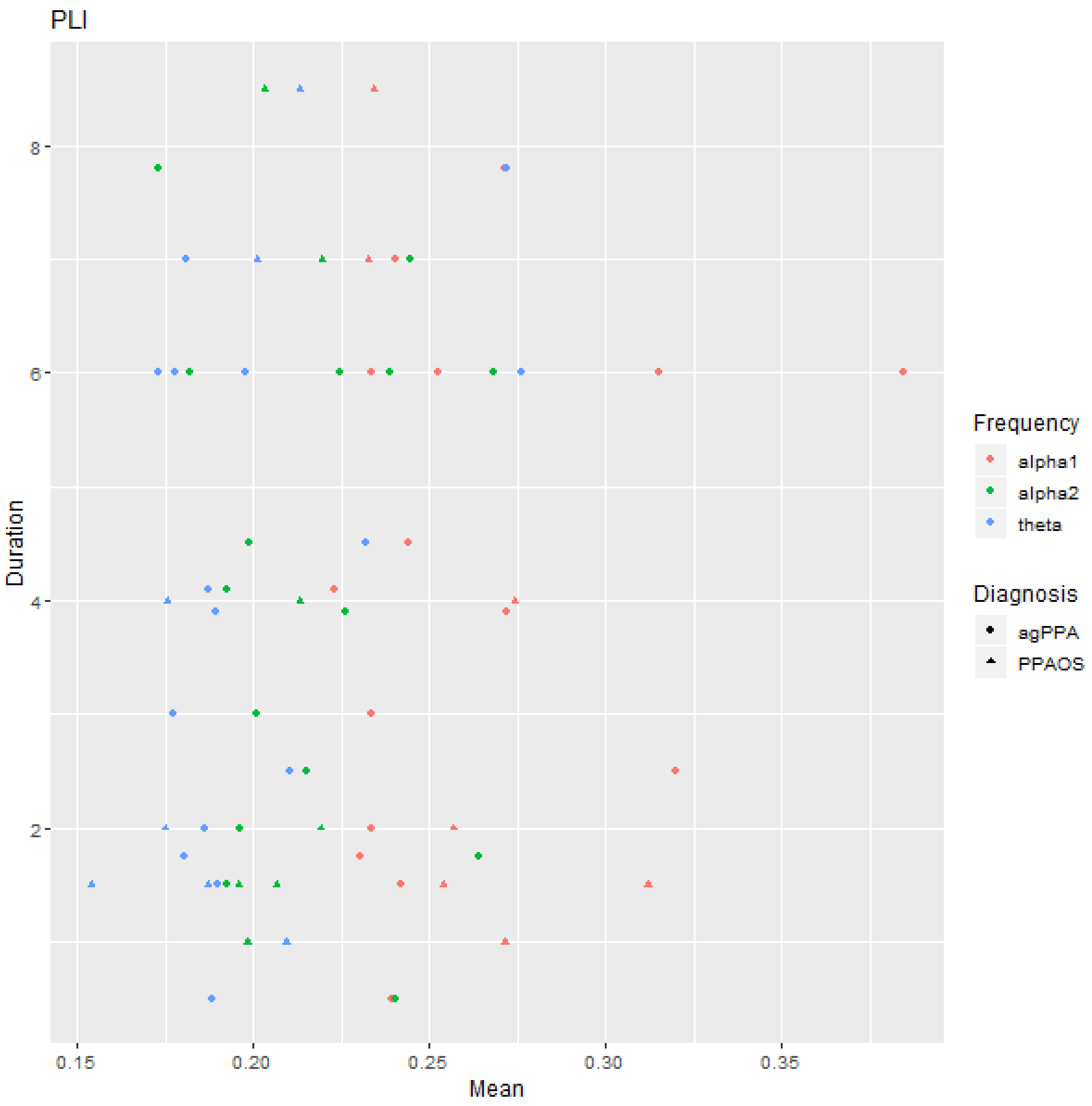

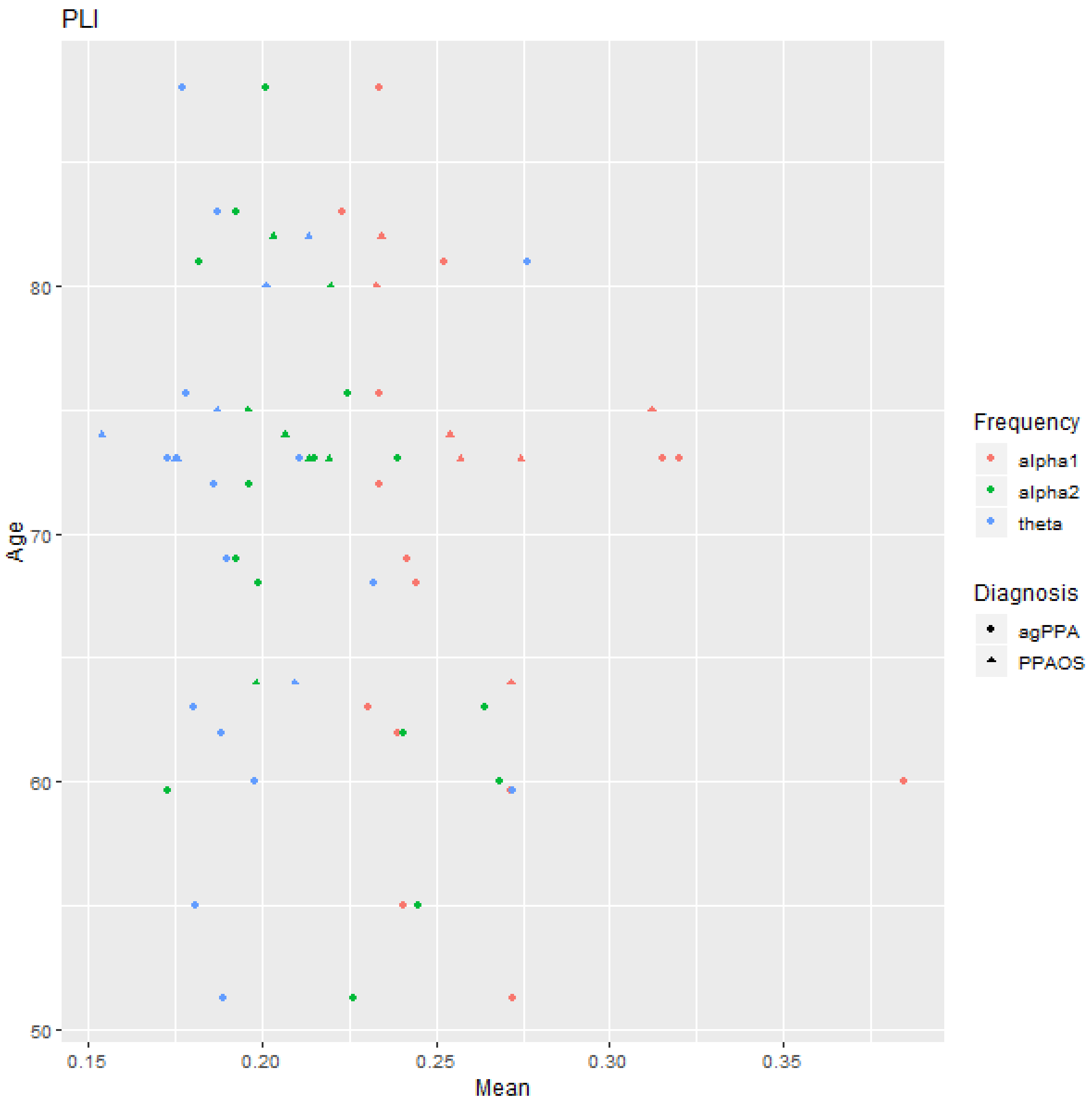

PLI
Age
80
70
60
50
0.15
0.20
0.25
0.30
0.35
Mean
Frequency
alpha1
alpha2
theta
Diagnosis
agPPA
PPAOS

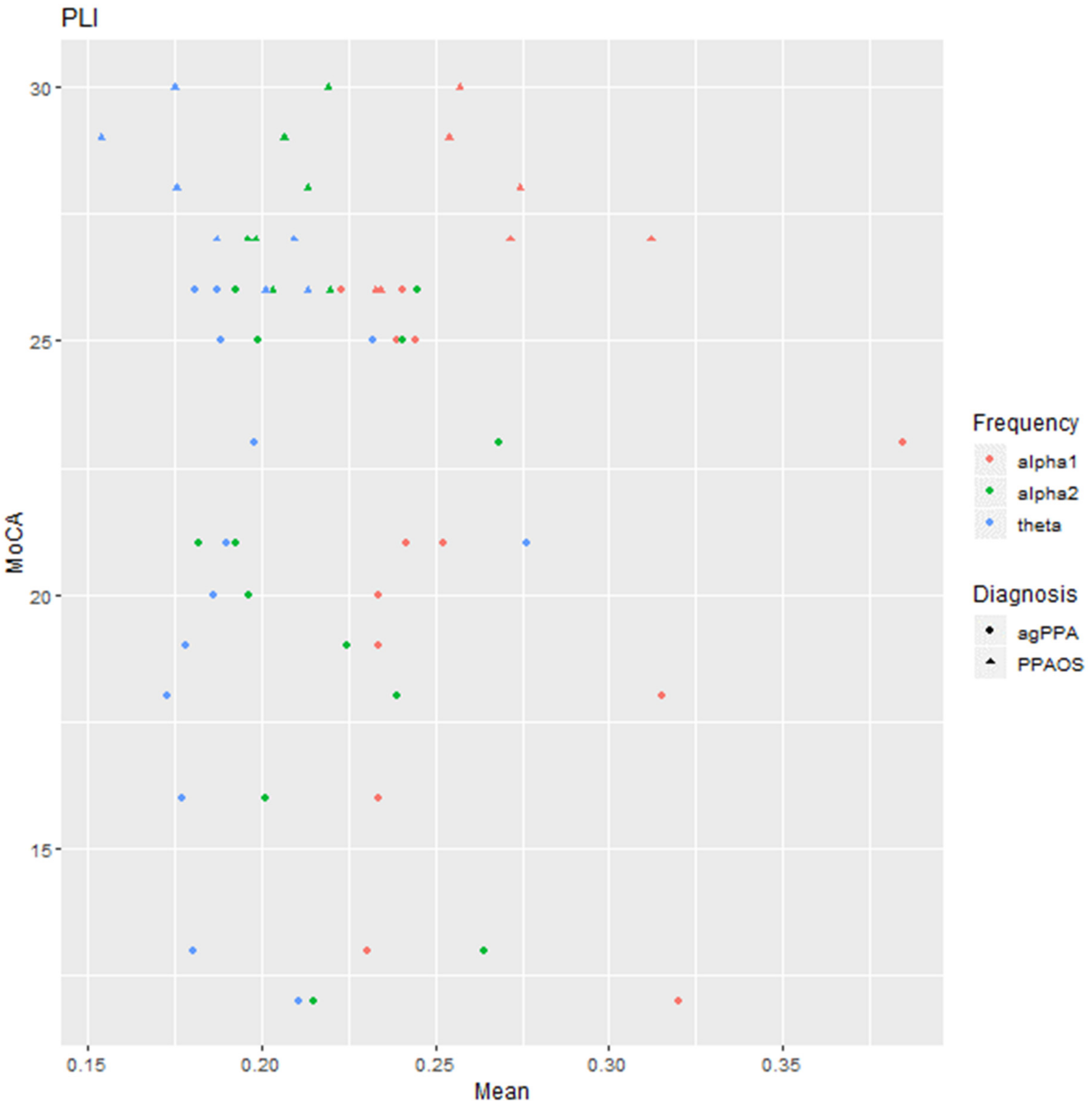
PLI
MoCA
Mean
30
25
20
15
0.15
0.20
0.25
0.30
0.35
Frequency
alpha1
alpha2
theta
Diagnosis
agPPA
PPAOS

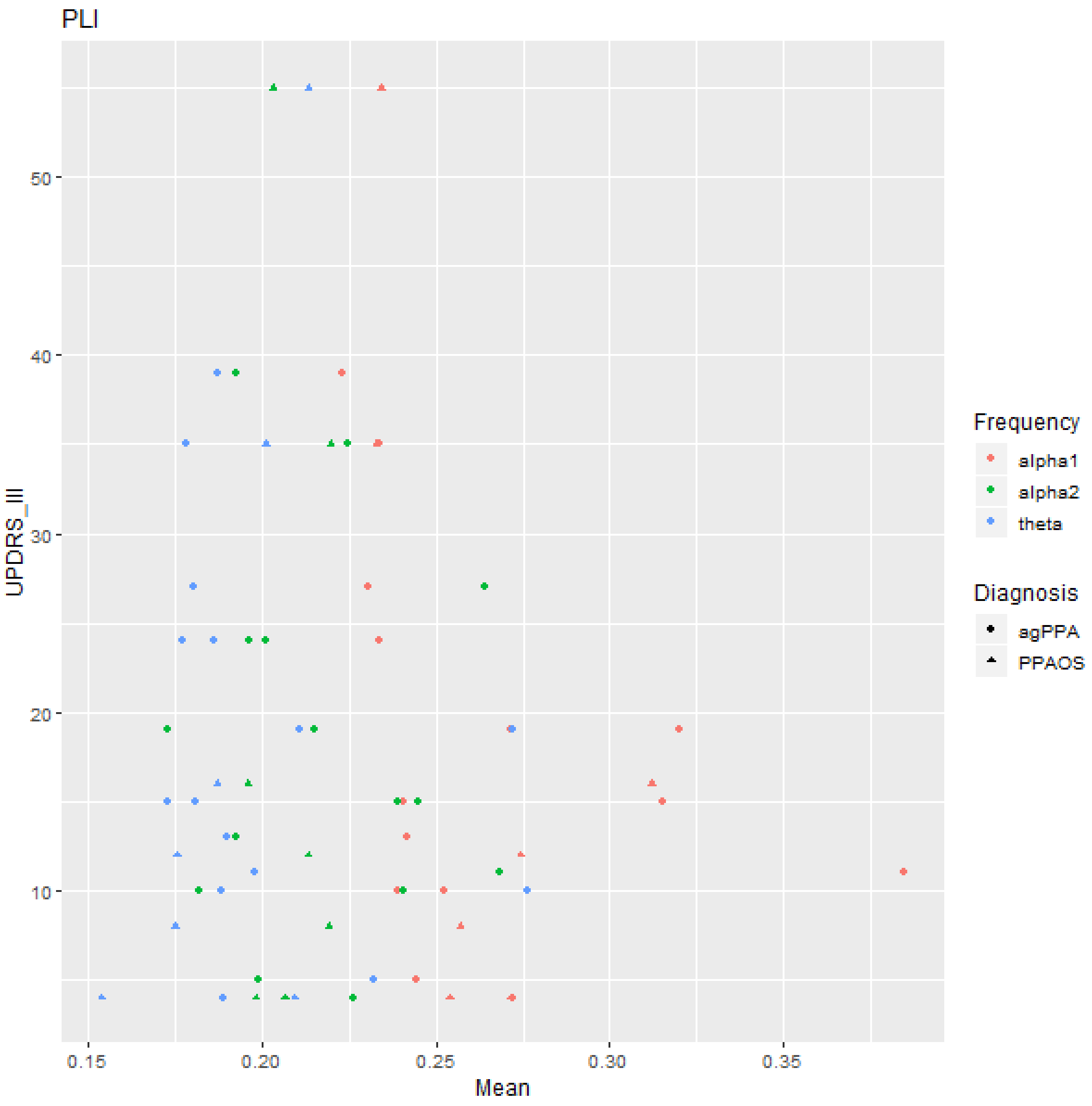

PLI
UPDRS_III
50
40
30
20
10
0.15
0.20
0.25
0.30
0.35
Mean
Frequency
alpha1
alpha2
theta
Diagnosis
agPPA
PPAOS

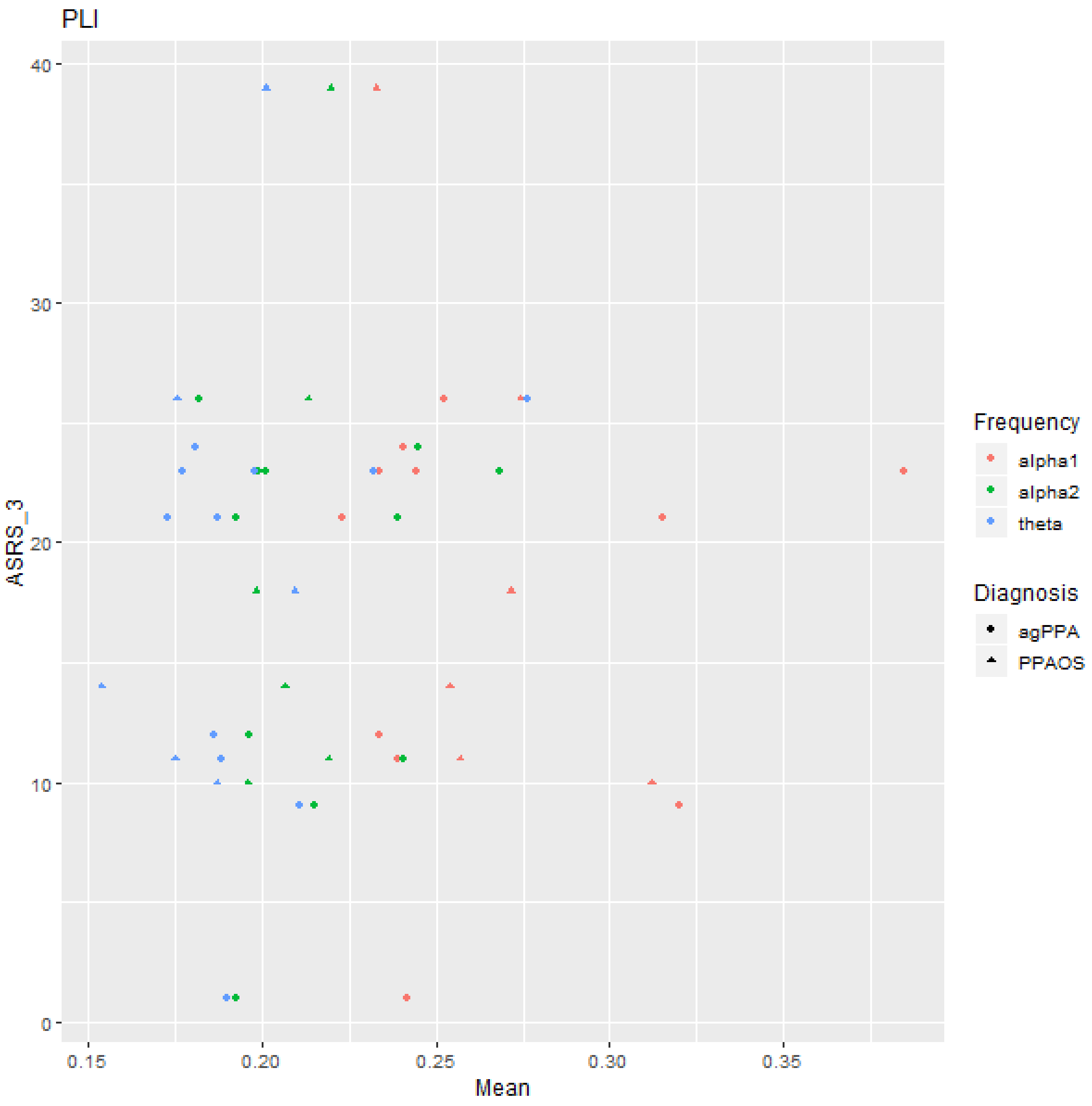

PLI
40
30
20
10
0
ASRS_3
0.15
0.20
0.25
0.30
0.35
Mean
Frequency
alpha1
alpha2
theta
Diagnosis
agPPA
PPAOS

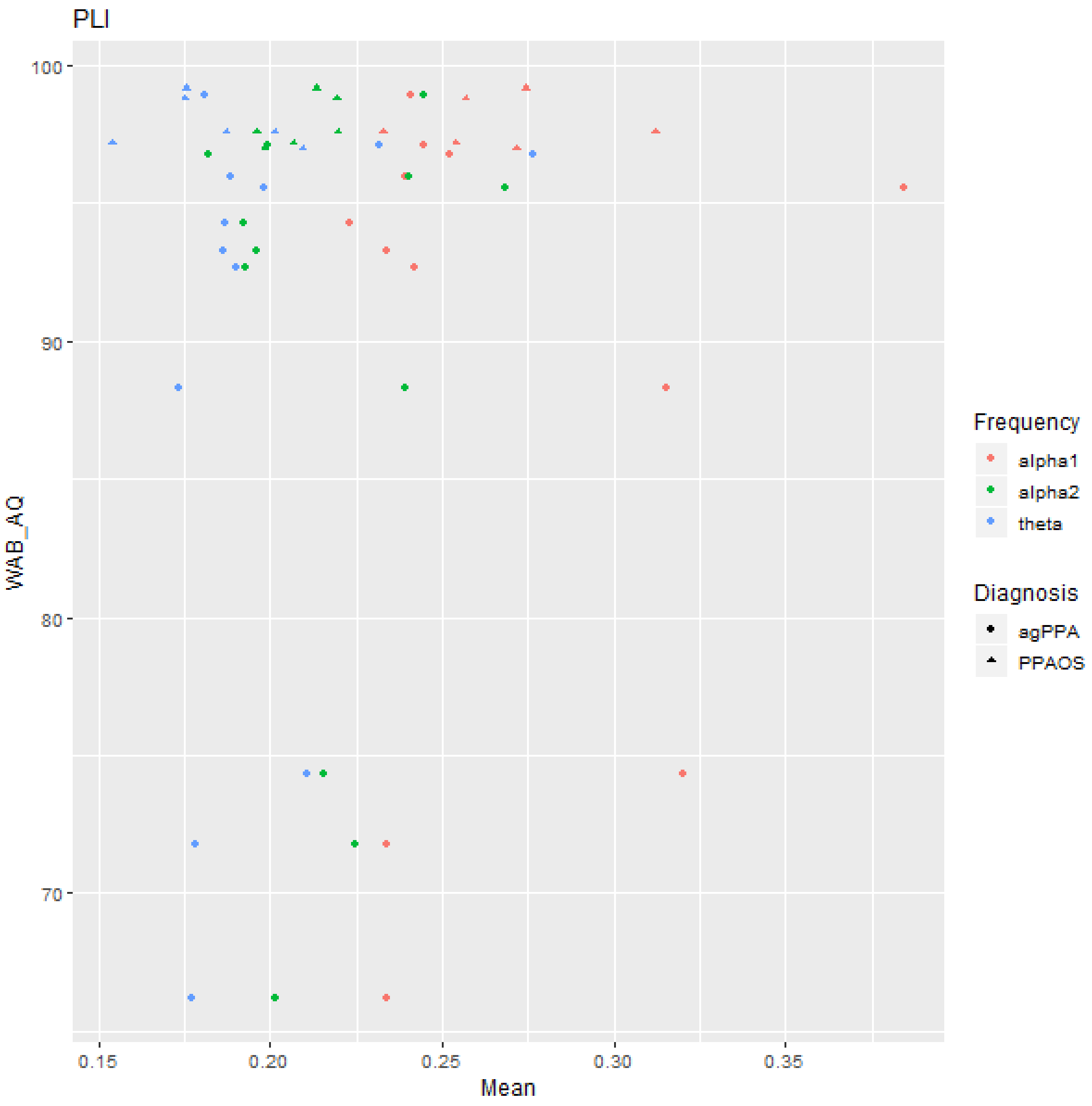

PLI
WAB_AQ
100
90
80
70
0.15
0.20
0.25
0.30
0.35
Mean
Frequency
alpha1
alpha2
theta
Diagnosis
agPPA
PPAOS

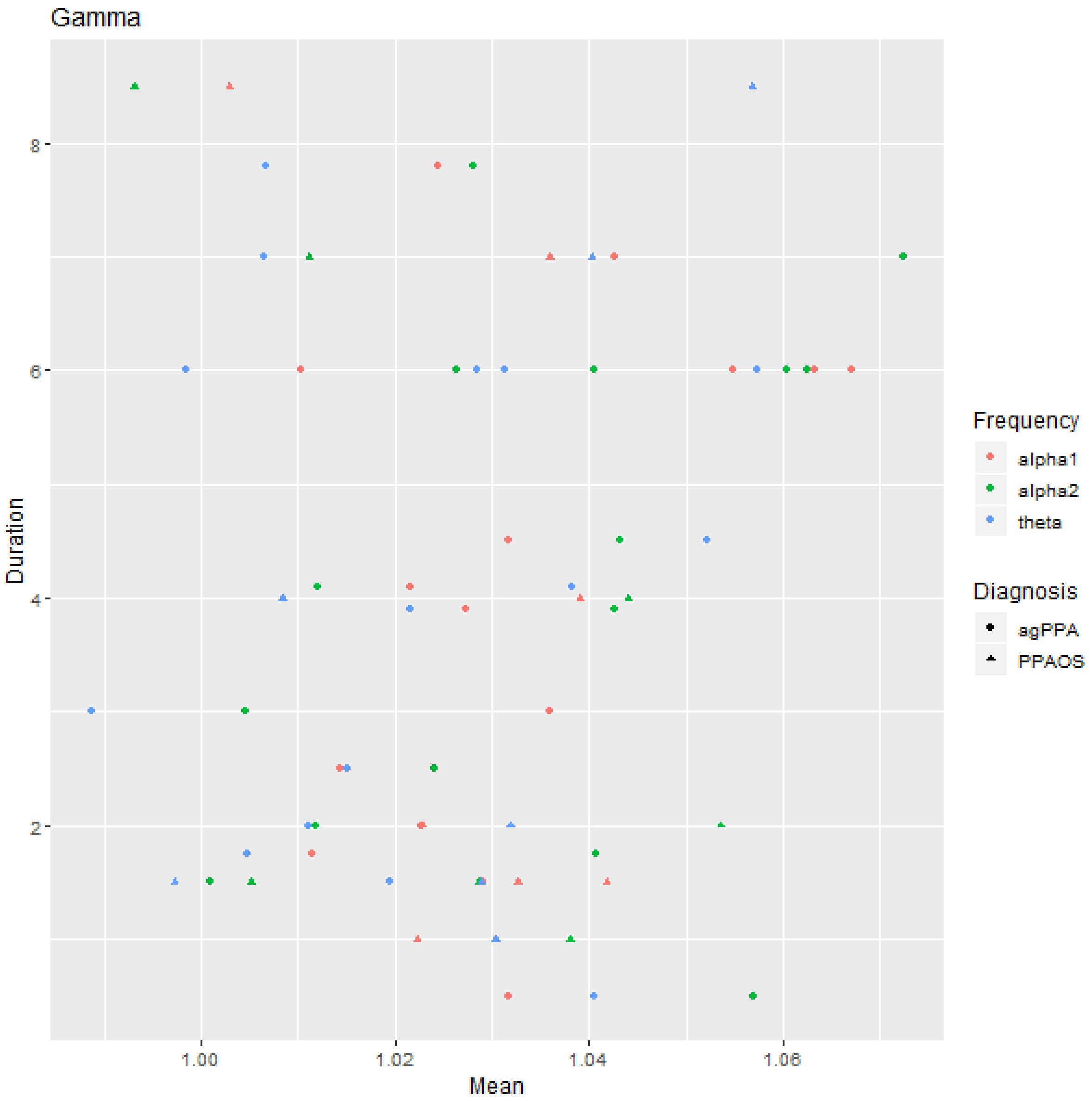
Gamma
Duration
8
6
4
2
1.00
1.02
1.04
1.06
Mean
Frequency
alpha1
alpha2
theta
Diagnosis
agPPA
PPAOS

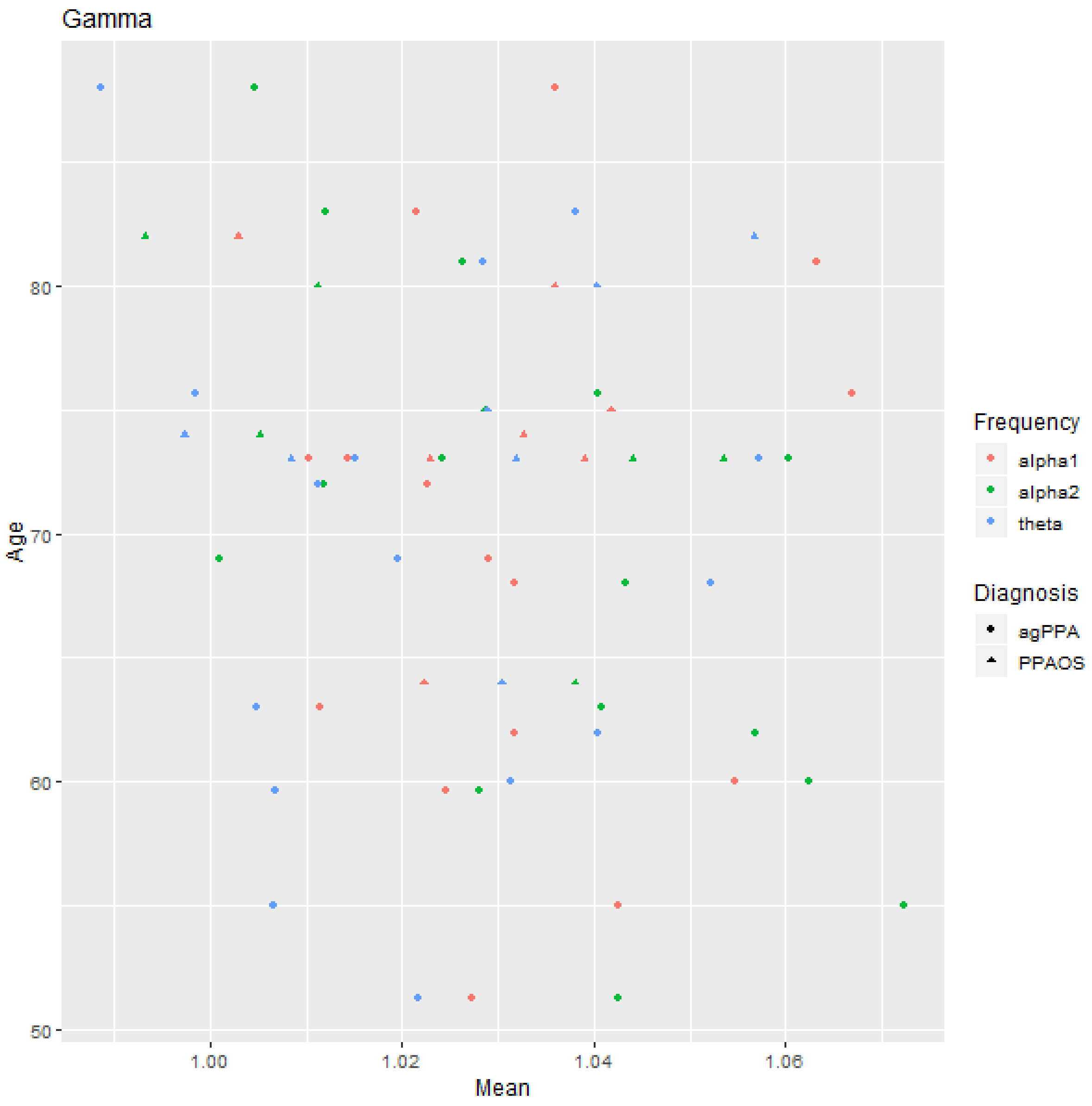

Gamma
Age
80
70
60
50
1.00
1.02
1.04
1.06
Mean
Frequency
alpha1
alpha2
theta
Diagnosis
agPPA
PPAOS

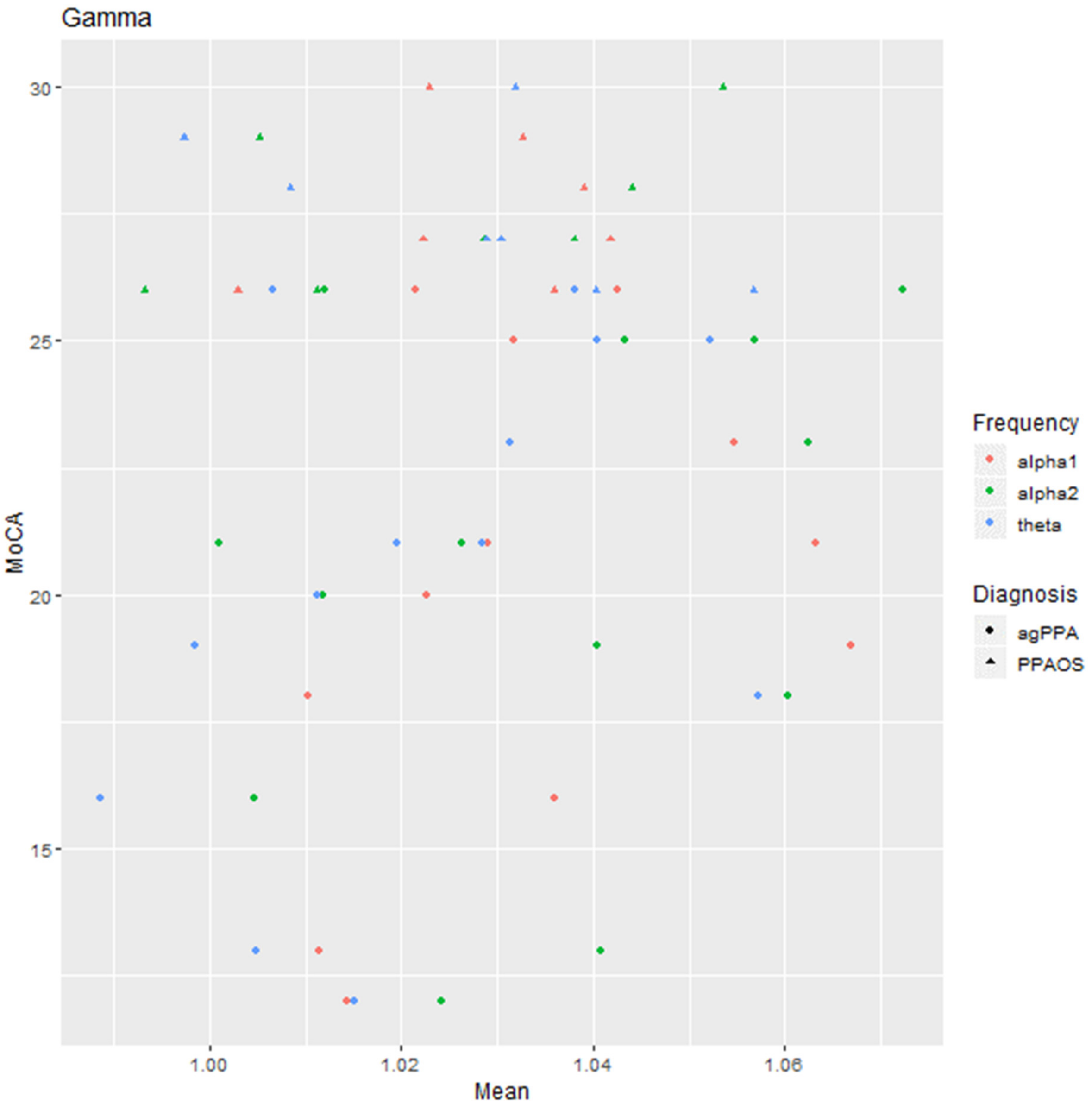

Gamma
30
25
20
15
MoCA
1.00
1.02
1.04
1.06
Mean
Frequency
alpha1
alpha2
theta
Diagnosis
agPPA
PPAOS

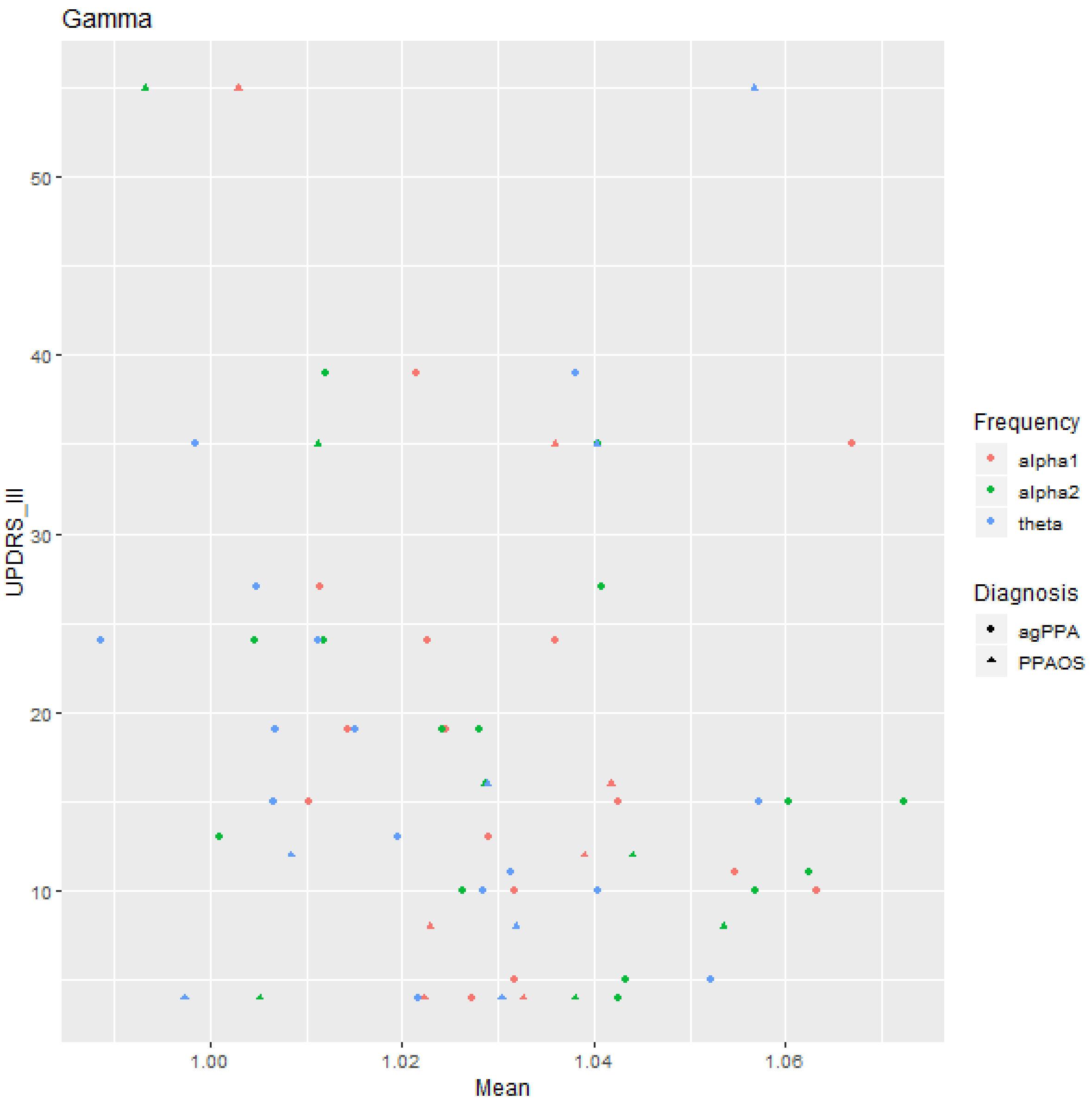
Gamma
UPDRS_III
10
20
30
40
50
1.00
1.02
1.04
1.06
Mean
Frequency
alpha1
alpha2
theta
Diagnosis
agPPA
PPAOS

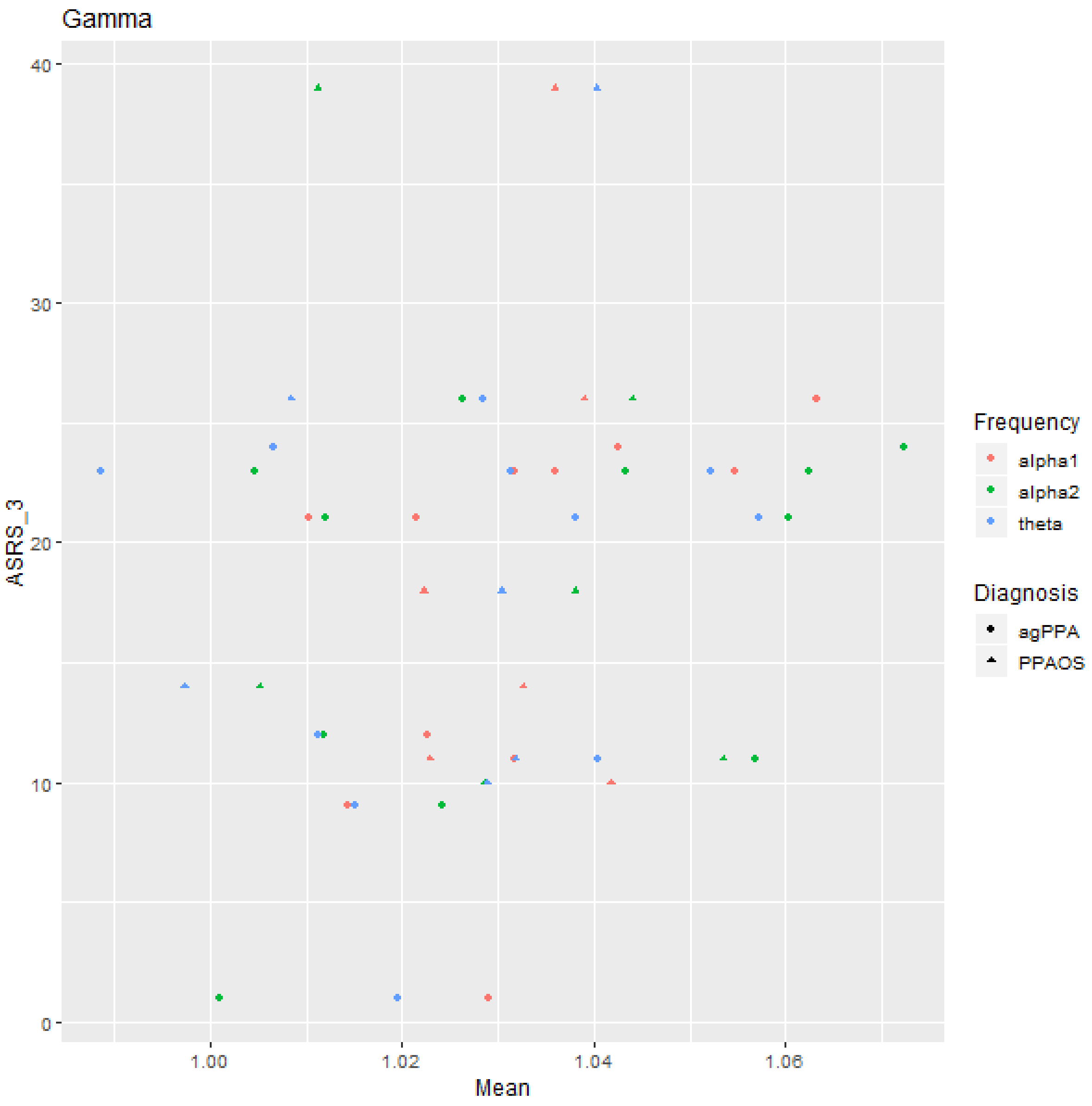
Gamma
40
30
20
10
0
ASRS_3
1.00
1.02
1.04
1.06
Mean
Frequency
alpha1
alpha2
theta
Diagnosis
agPPA
PPAOS

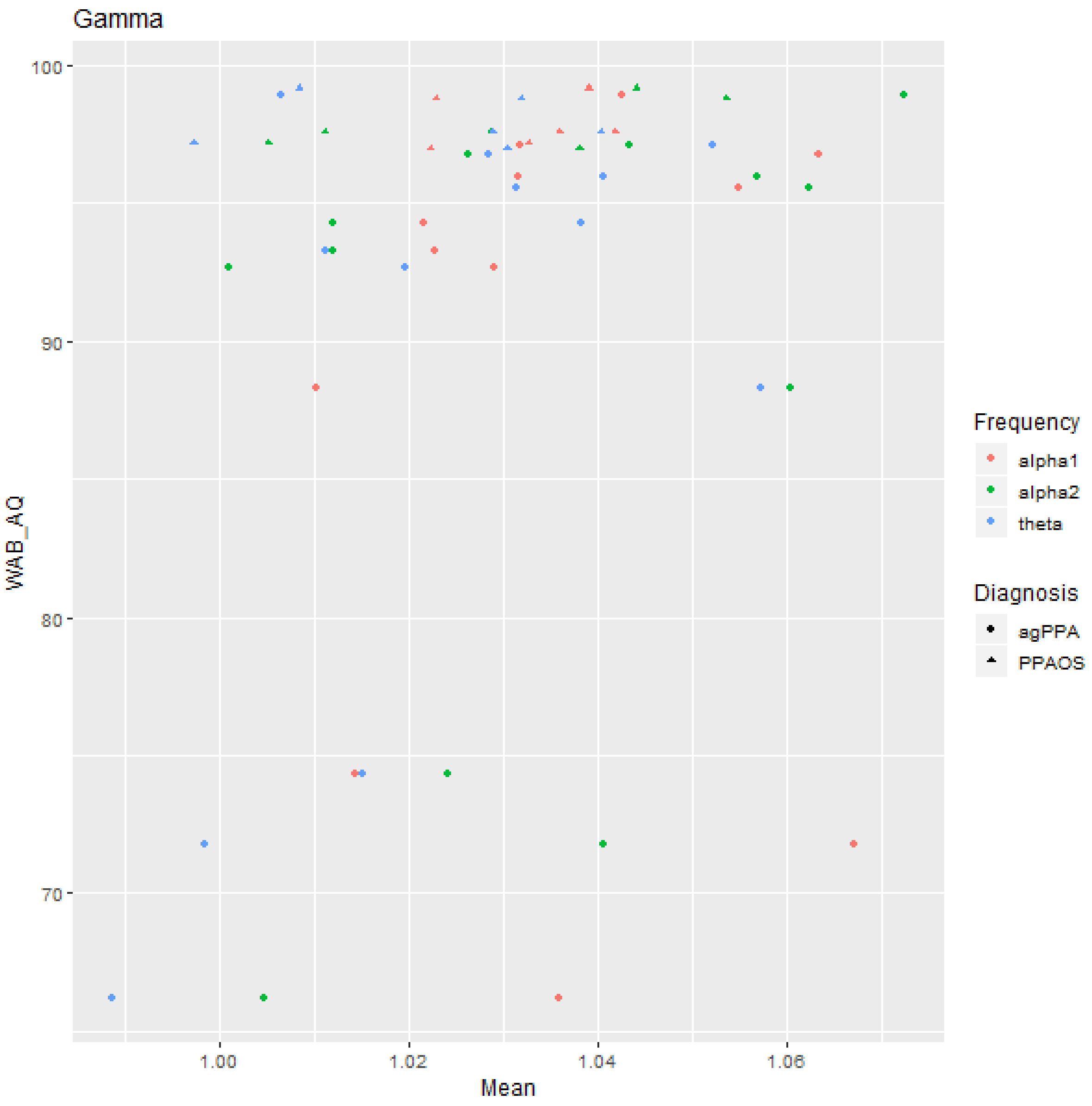
Gamma
100
90
80
70
WAB_AQ
1.00
1.02
1.04
1.06
Mean
Frequency
alpha1
alpha2
theta
Diagnosis
agPPA
PPAOS

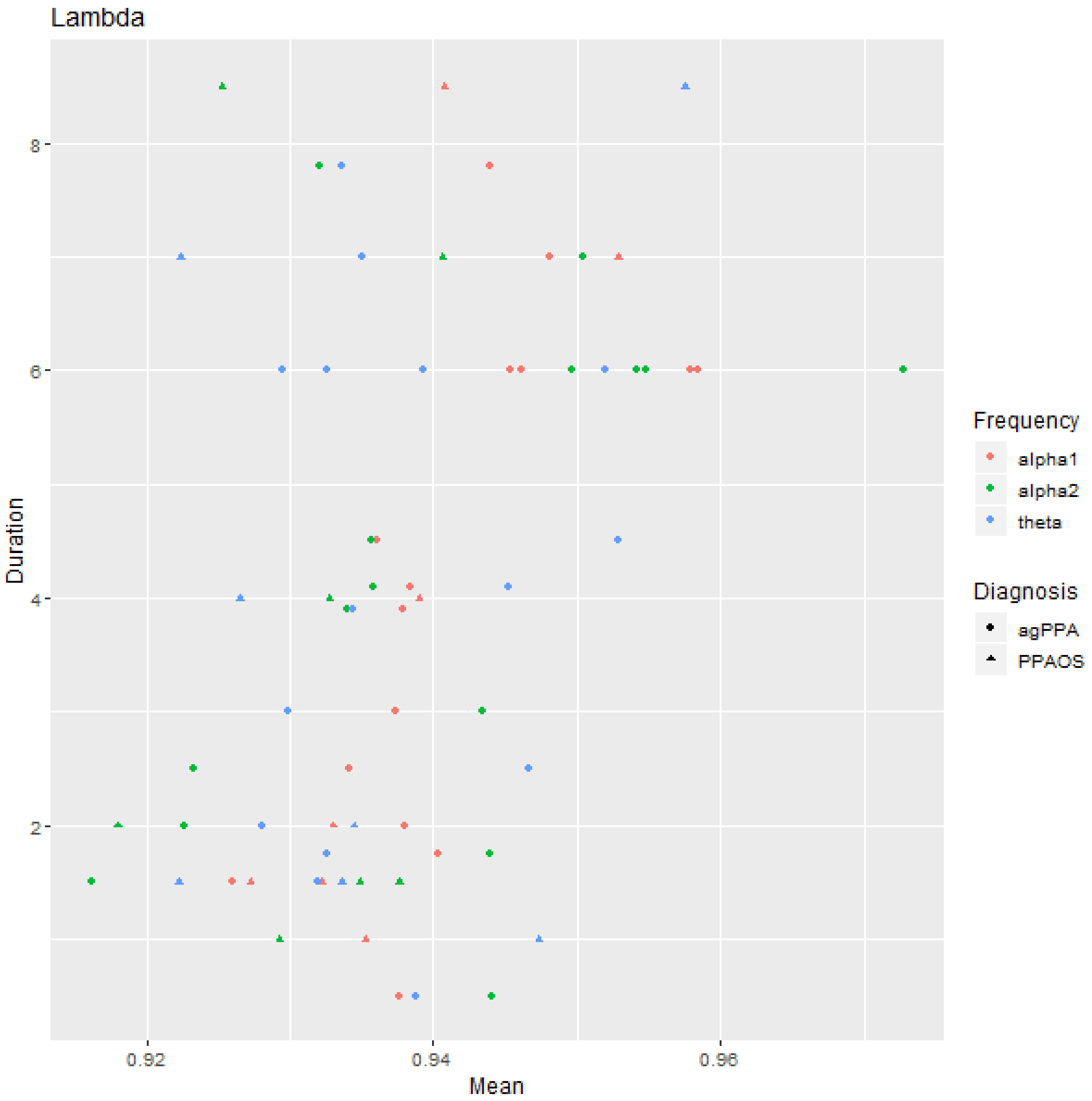

Lambda
Duration
8
6
4
2
0.92
0.94
0.96
Mean
Frequency
alpha1
alpha2
theta
Diagnosis
agPPA
PPAOS

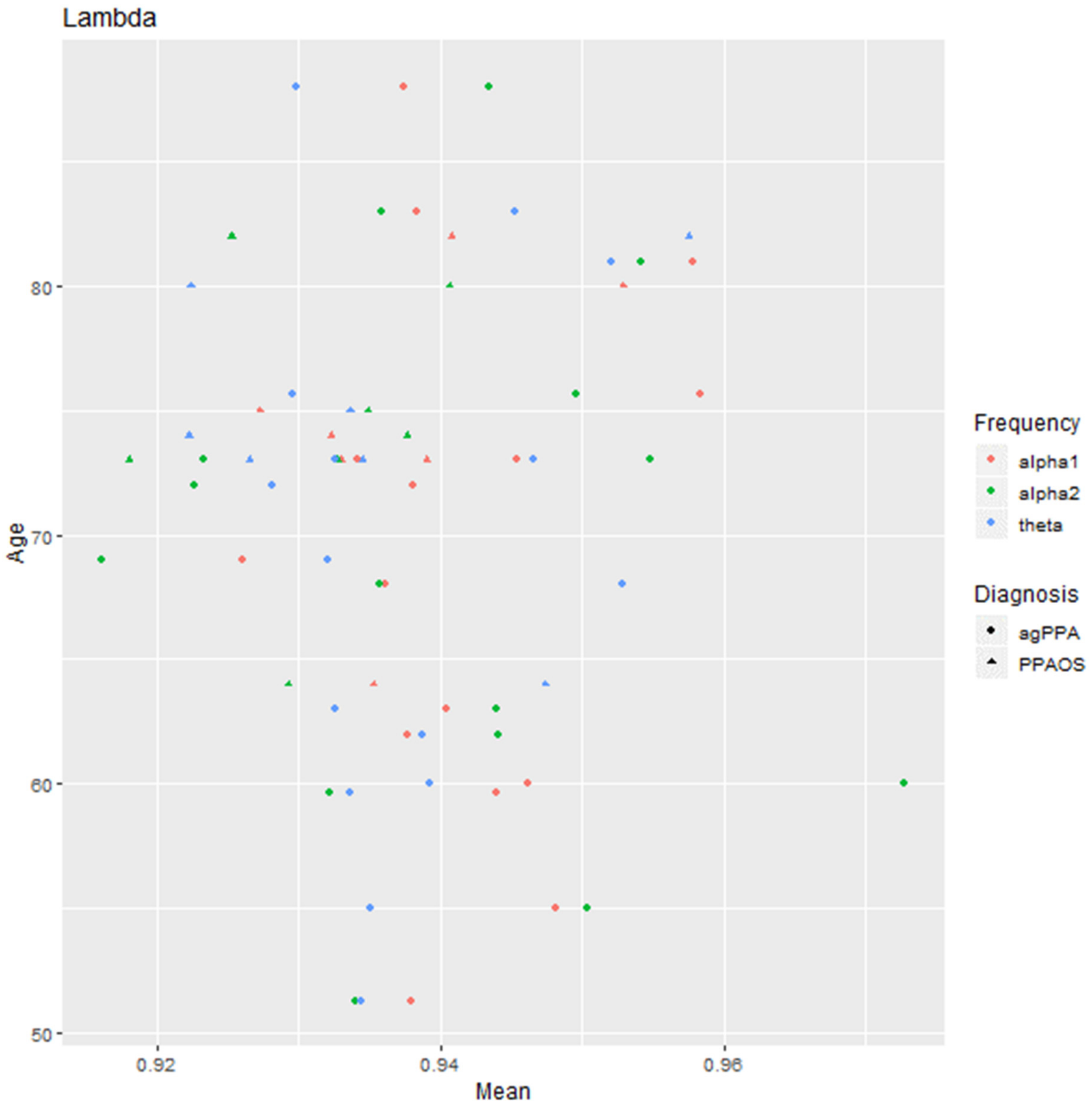
Lambda
Age
80
70
60
50
0.92
0.94
0.96
Mean
Frequency
alpha1
alpha2
theta
Diagnosis
agPPA
PPAOS

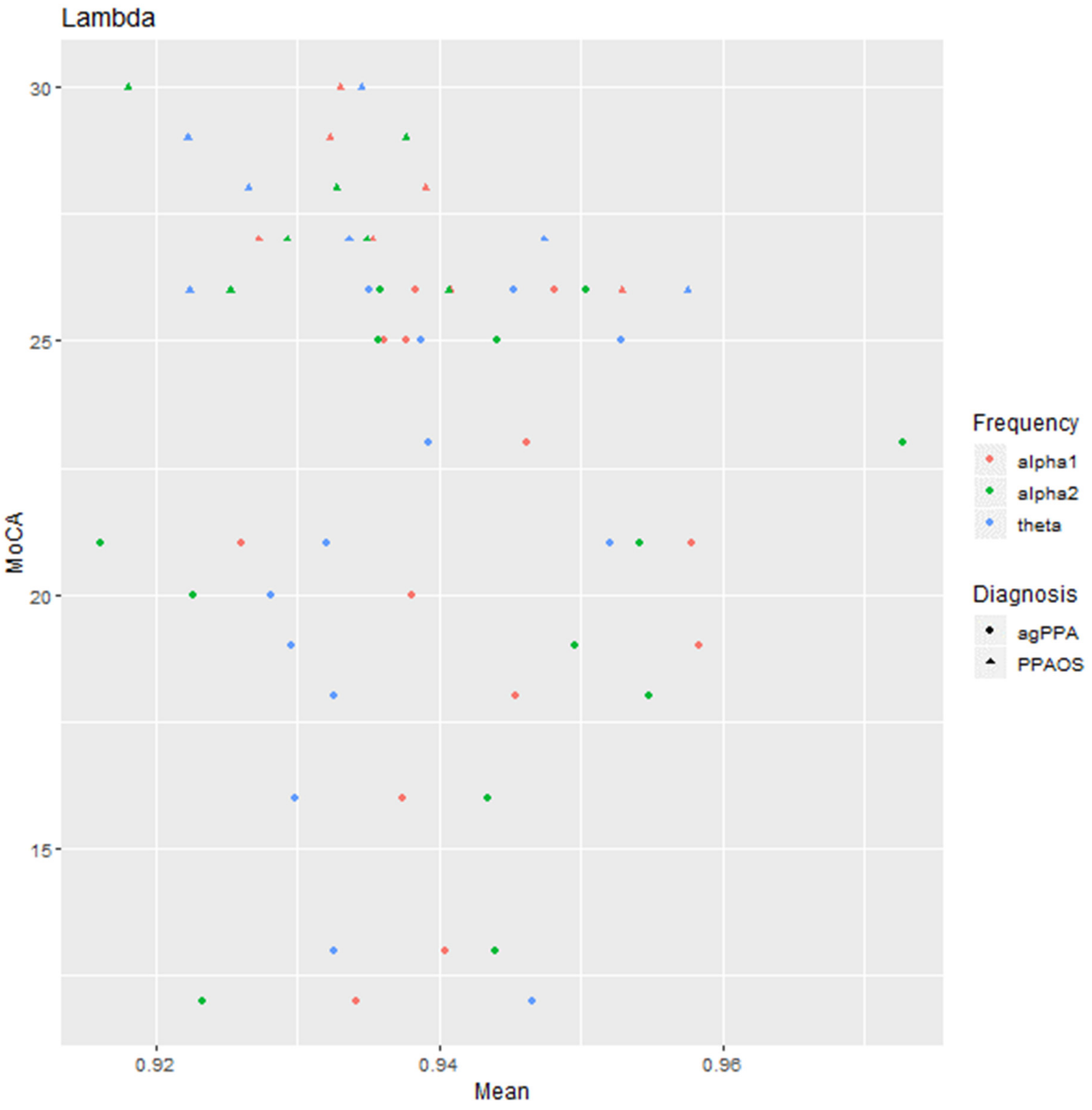

Lambda
30
25
20
15
MoCA
0.92
0.94
0.96
Mean
Frequency
alpha1
alpha2
theta
Diagnosis
agPPA
PPAOS

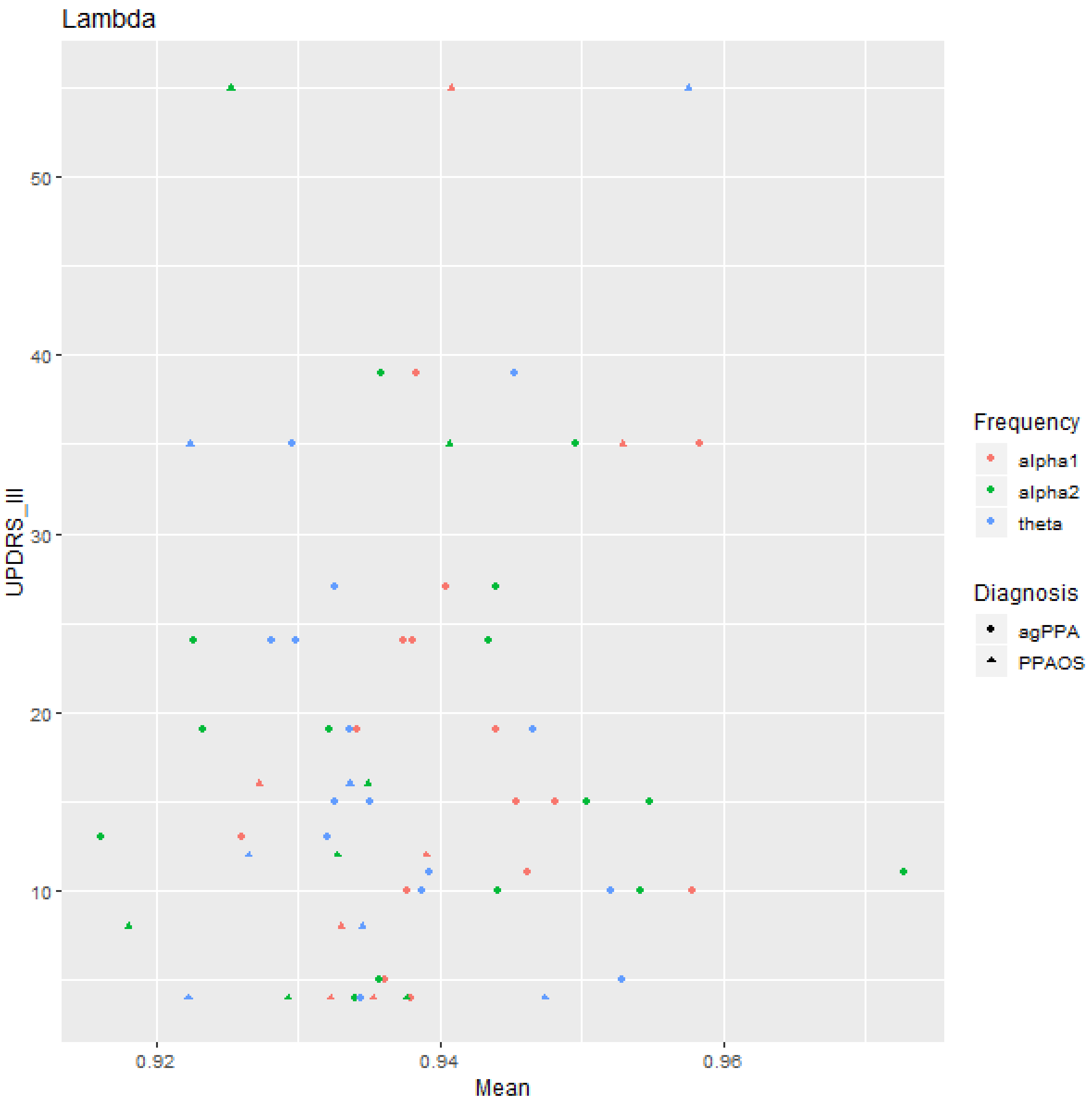

Lambda
UPDRS_III
50
40
30
20
10
0.92
0.94
0.96
Mean
Frequency
alpha1
alpha2
theta
Diagnosis
agPPA
PPAOS

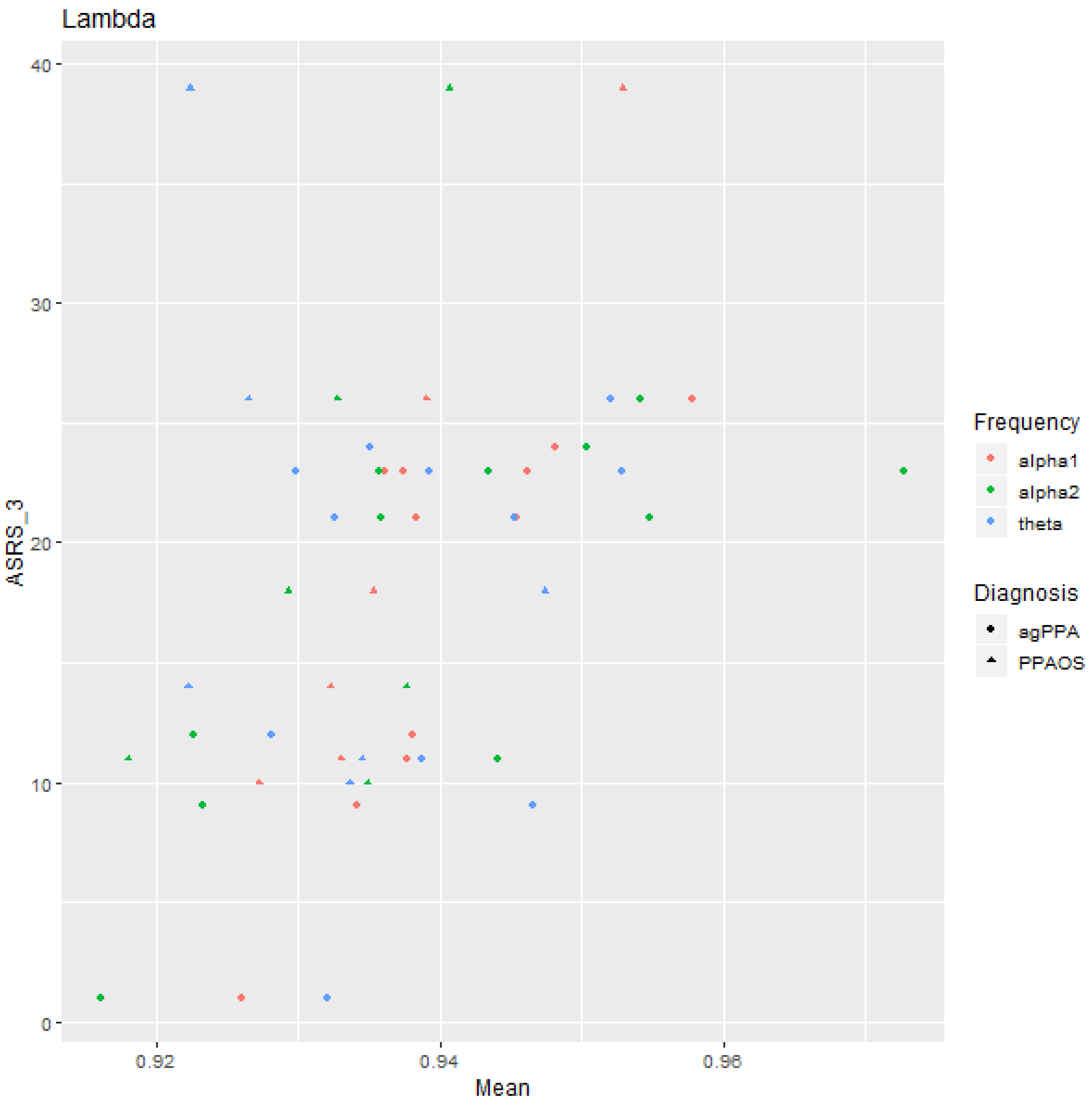
Lambda
ASRS_3
Mean
0
10
20
30
40
0.92
0.94
0.96
Frequency
alpha1
alpha2
theta
Diagnosis
agPPA
PPAOS

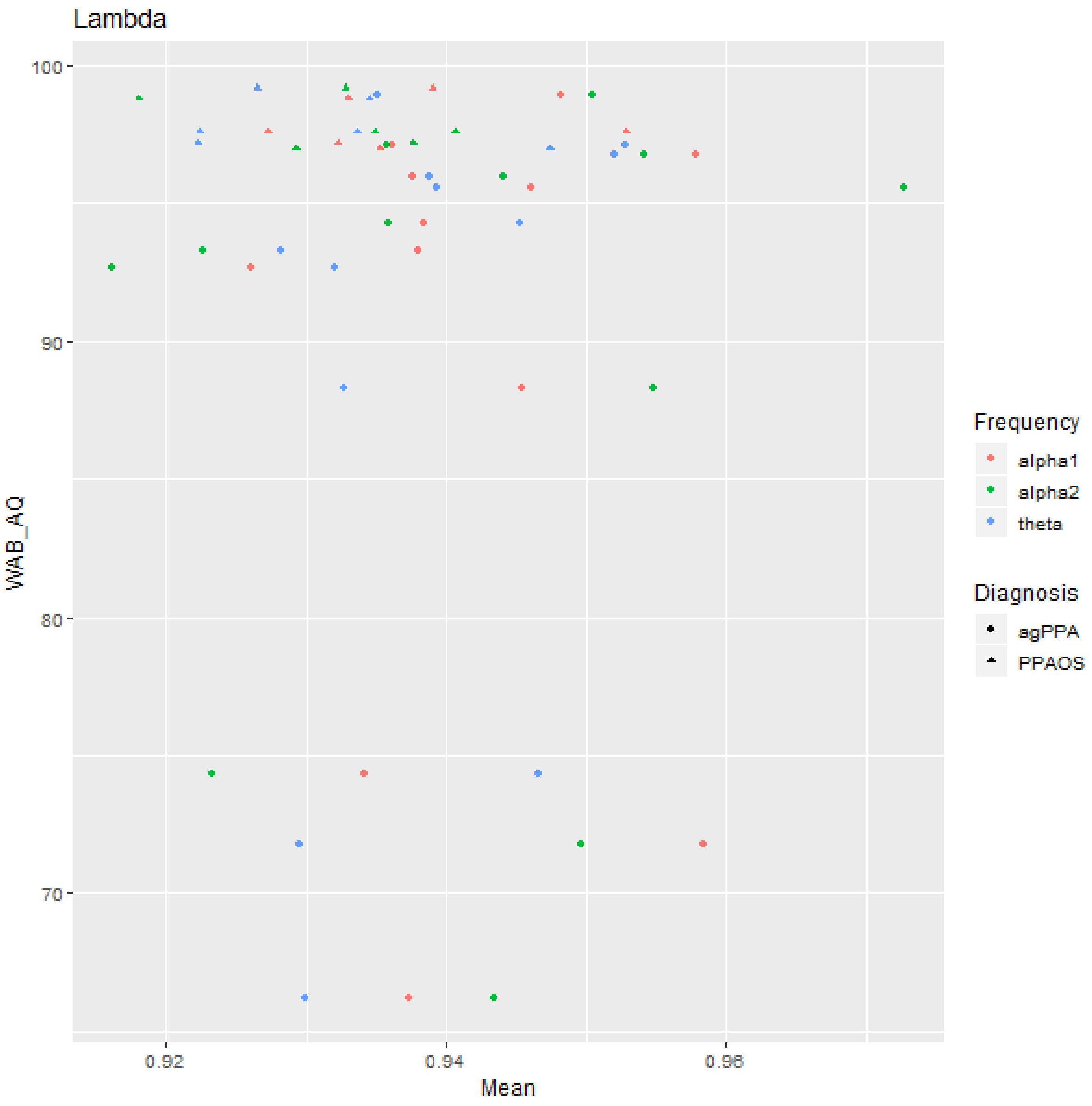

Lambda
100
90
80
70
WAB_AQ
0.92
0.94
0.96
Mean
Frequency
alpha1
alpha2
theta
Diagnosis
agPPA
PPAOS

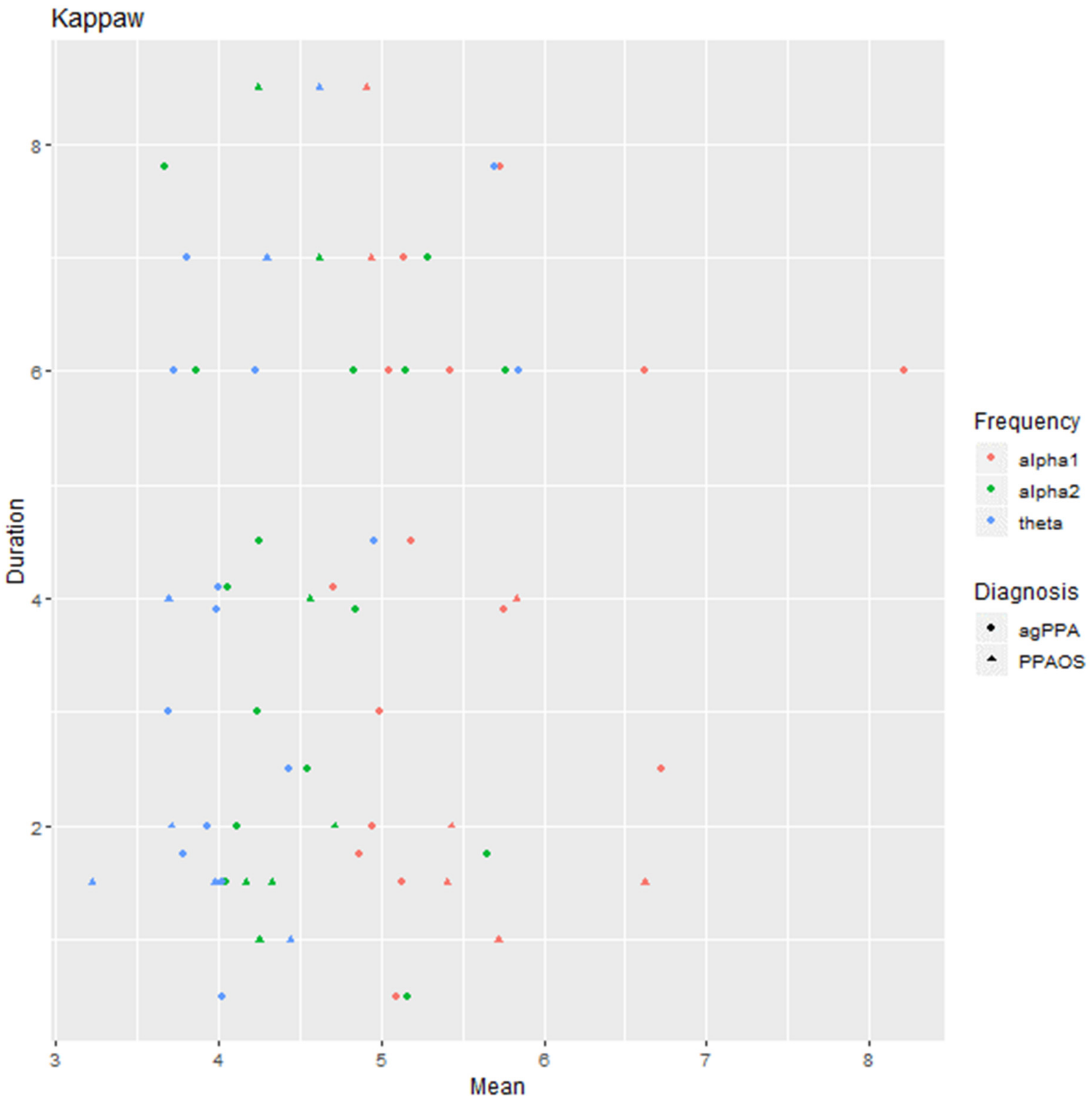

Kappaw
Duration
Mean
8
6
4
2
3
4
5
6
7
8
Frequency
alpha1
alpha2
theta
Diagnosis
agPPA
PPAOS

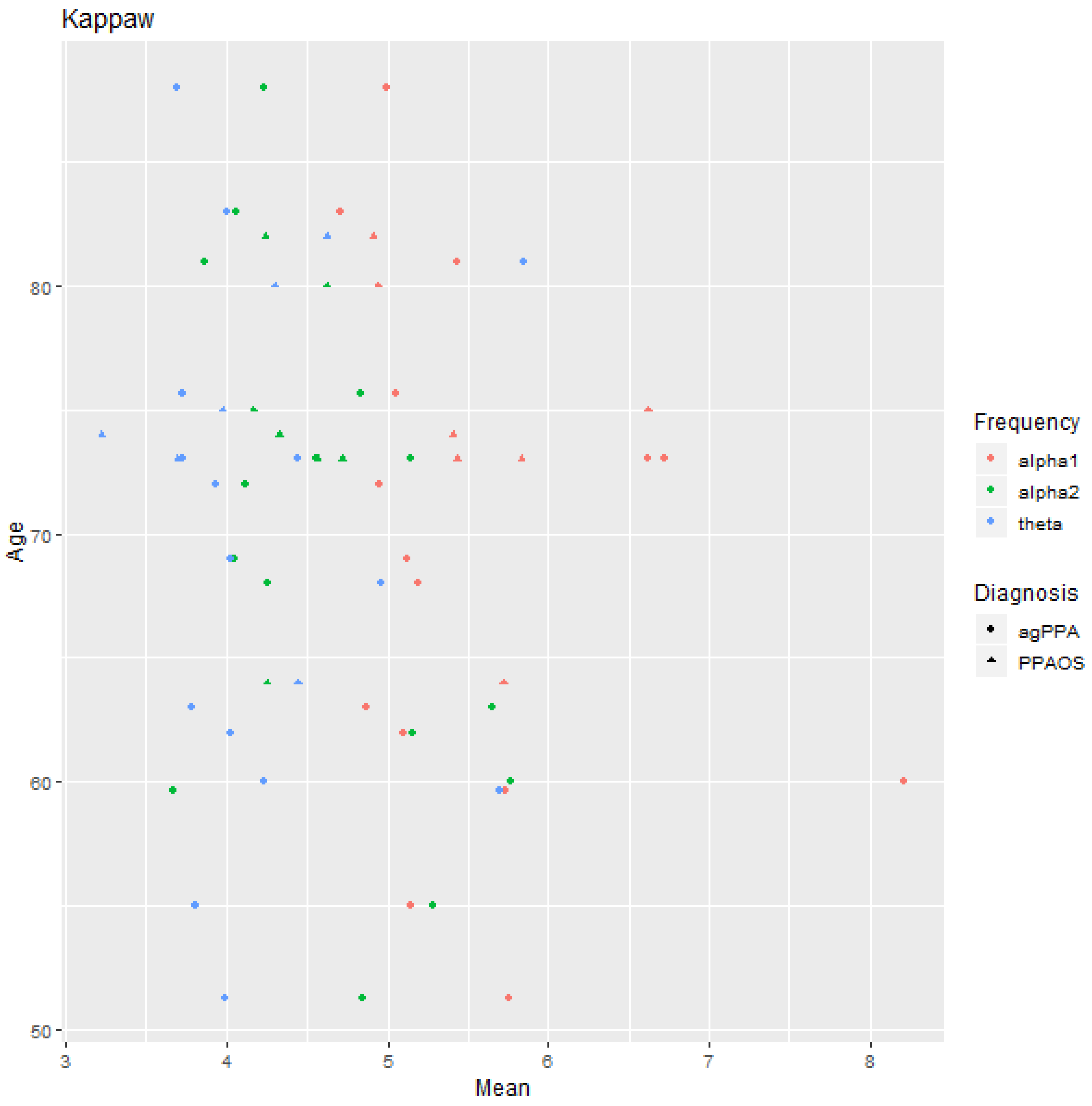
Kappaw
Age
Mean
80
70
60
50
3
4
5
6
7
8
Frequency
alpha1
alpha2
theta
Diagnosis
agPPA
PPAOS

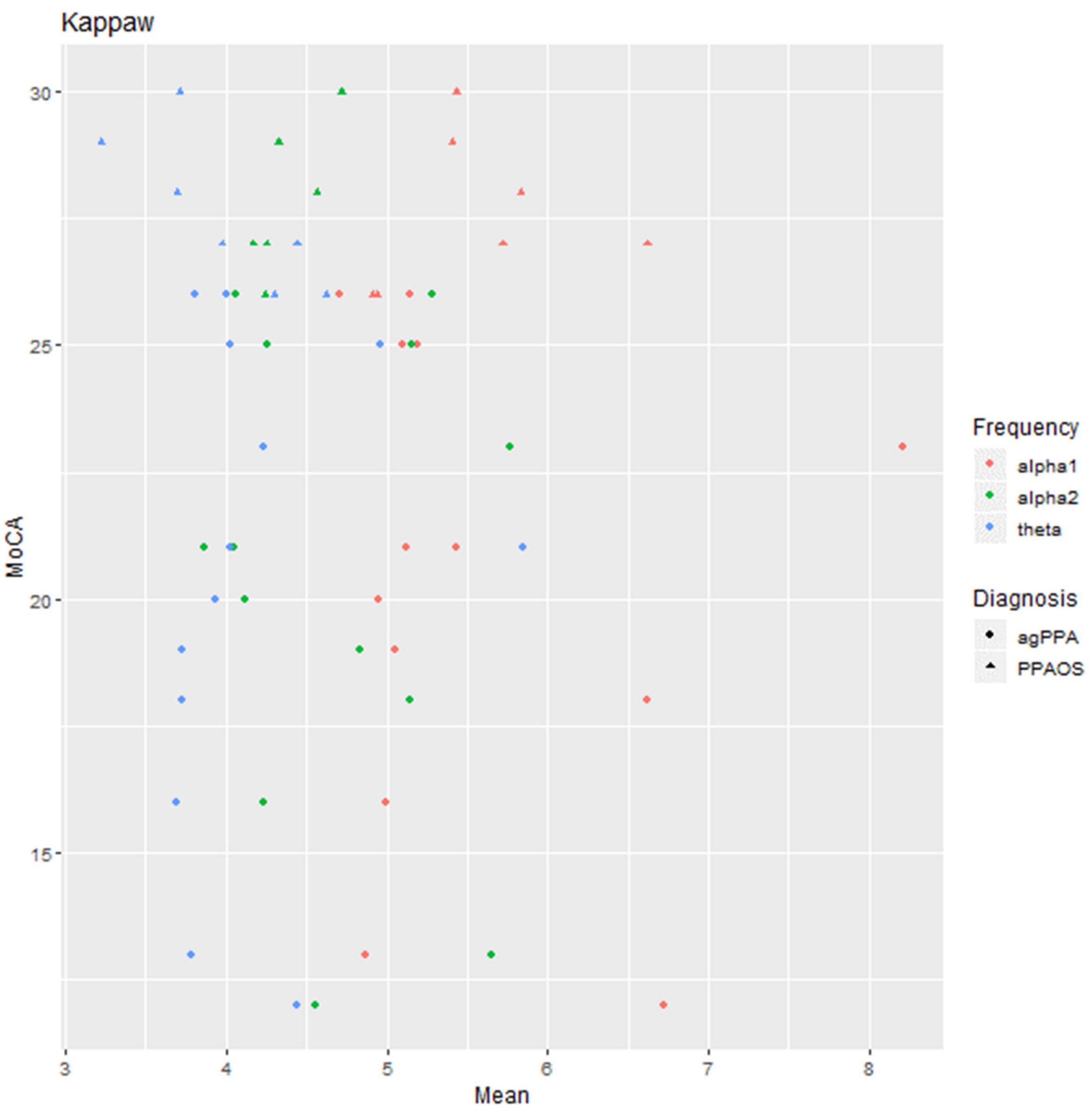

Kappaw
MoCA
Mean
30
25
20
15
3
4
5
6
7
8
Frequency
alpha1
alpha2
theta
Diagnosis
agPPA
PPAOS

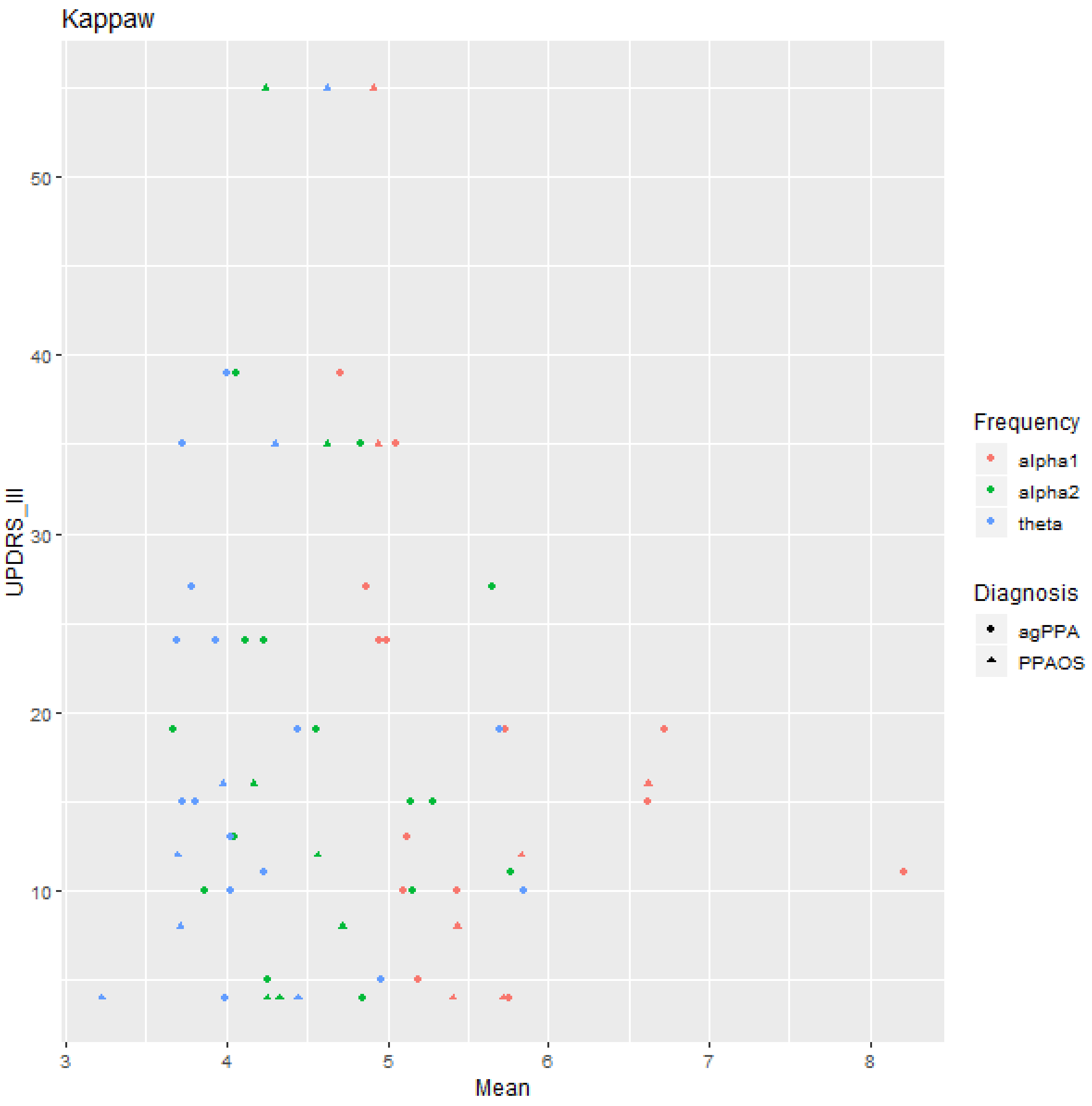

Kappaw
UPDRS_III
Mean
50
40
30
20
10
3
4
5
6
7
8
Frequency
alpha1
alpha2
theta
Diagnosis
agPPA
PPAOS

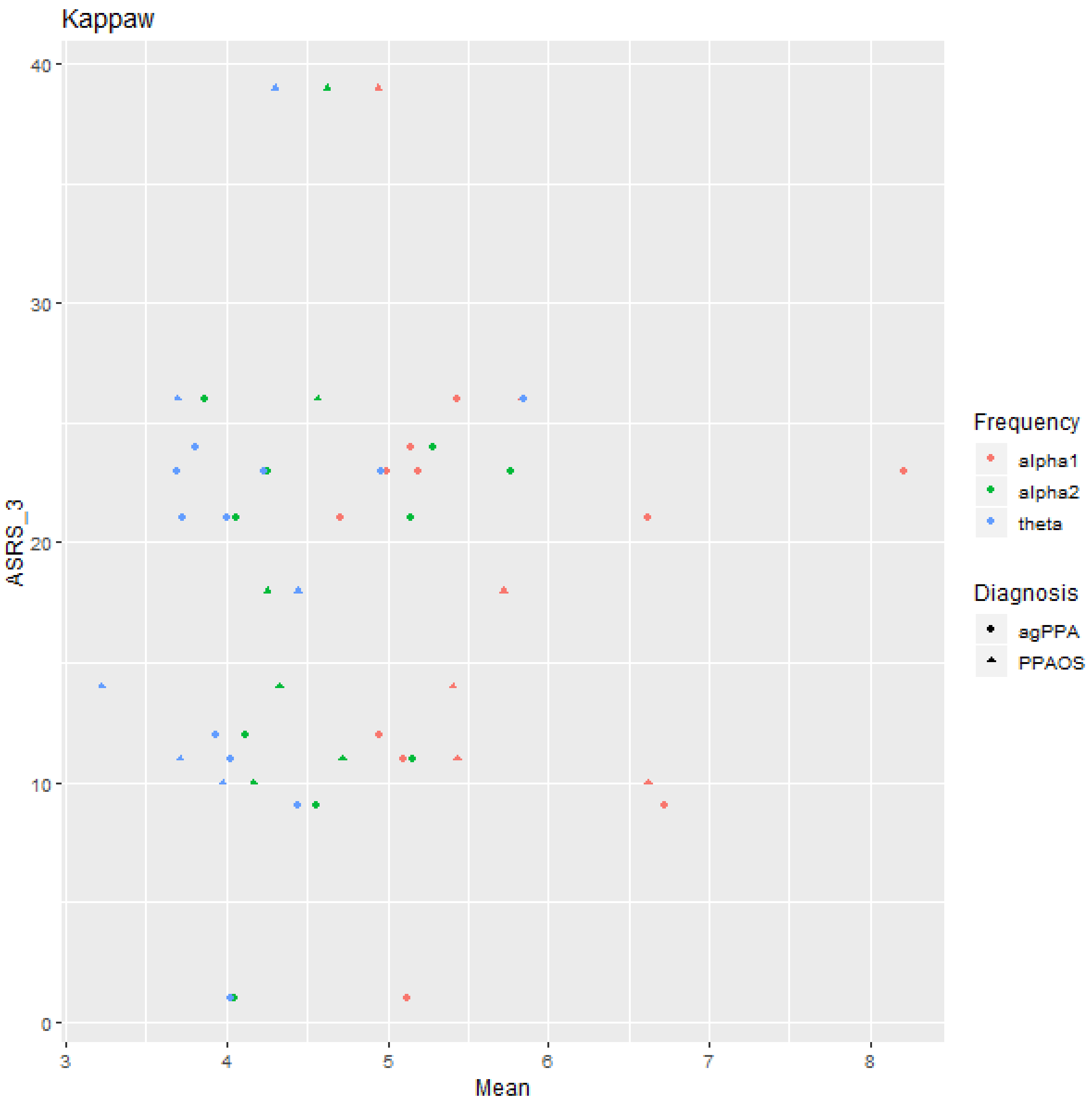

Kappaw
40
30
20
10
0
ASRS_3
3
4
5
6
7
8
Mean
Frequency
alpha1
alpha2
theta
Diagnosis
agPPA
PPAOS

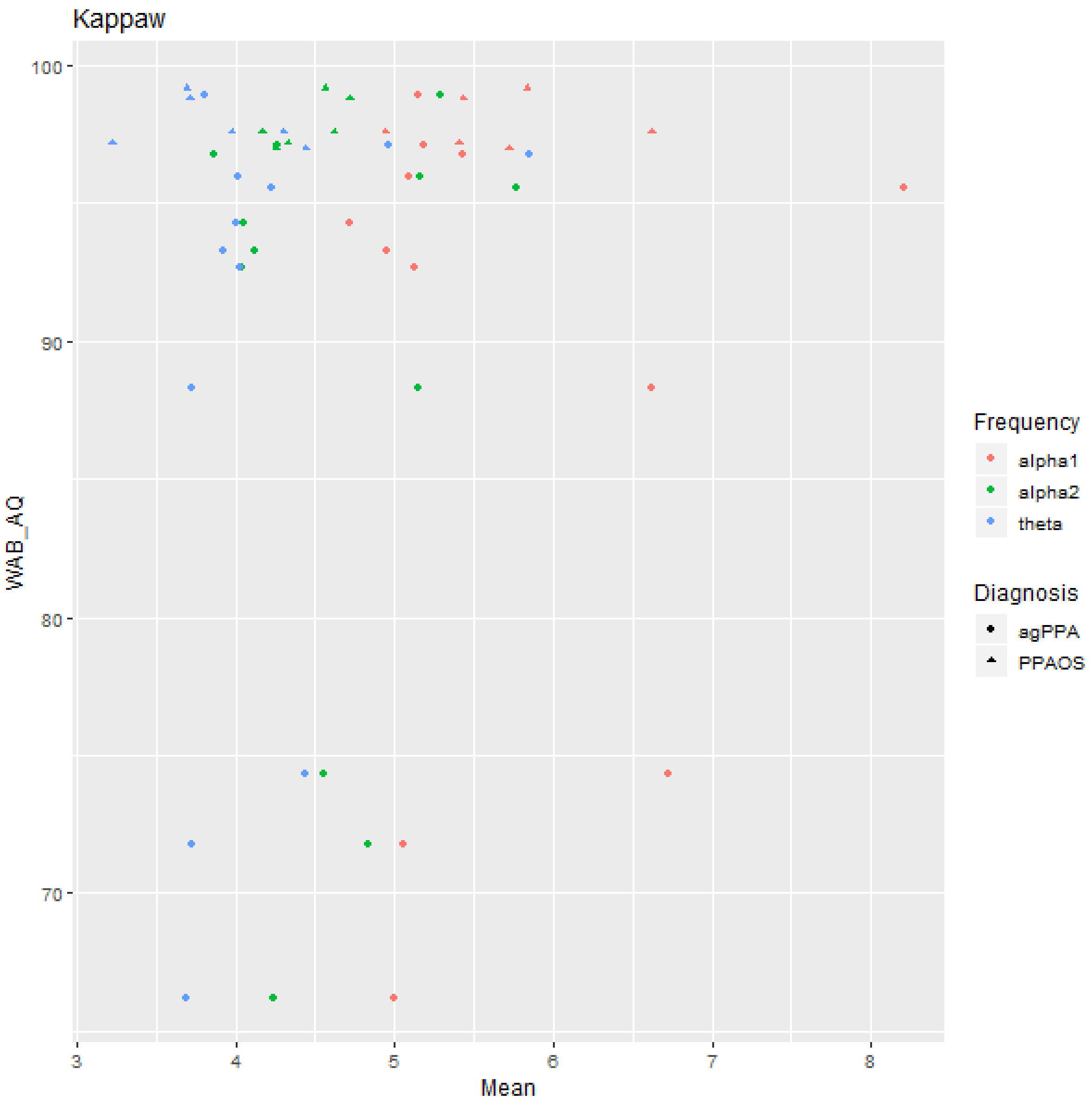

Kappaw
100
90
80
70
WAB_AQ
3
4
5
6
7
8
Mean
Frequency
alpha1
alpha2
theta
Diagnosis
agPPA
PPAOS

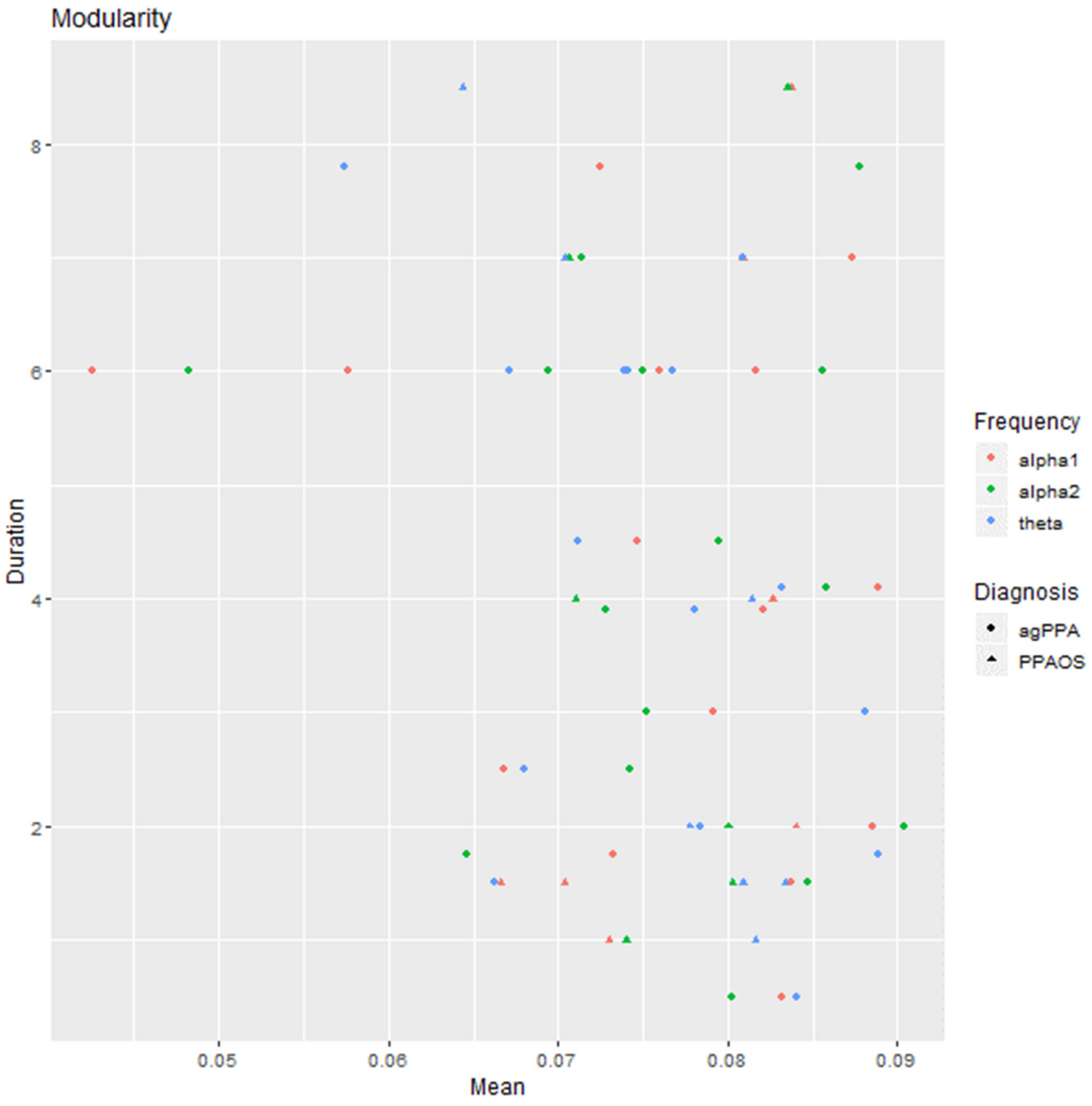

Modularity
Duration
8
6
4
2
Mean
0.05
0.06
0.07
0.08
0.09
Frequency
alpha1
alpha2
theta
Diagnosis
agPPA
PPAOS

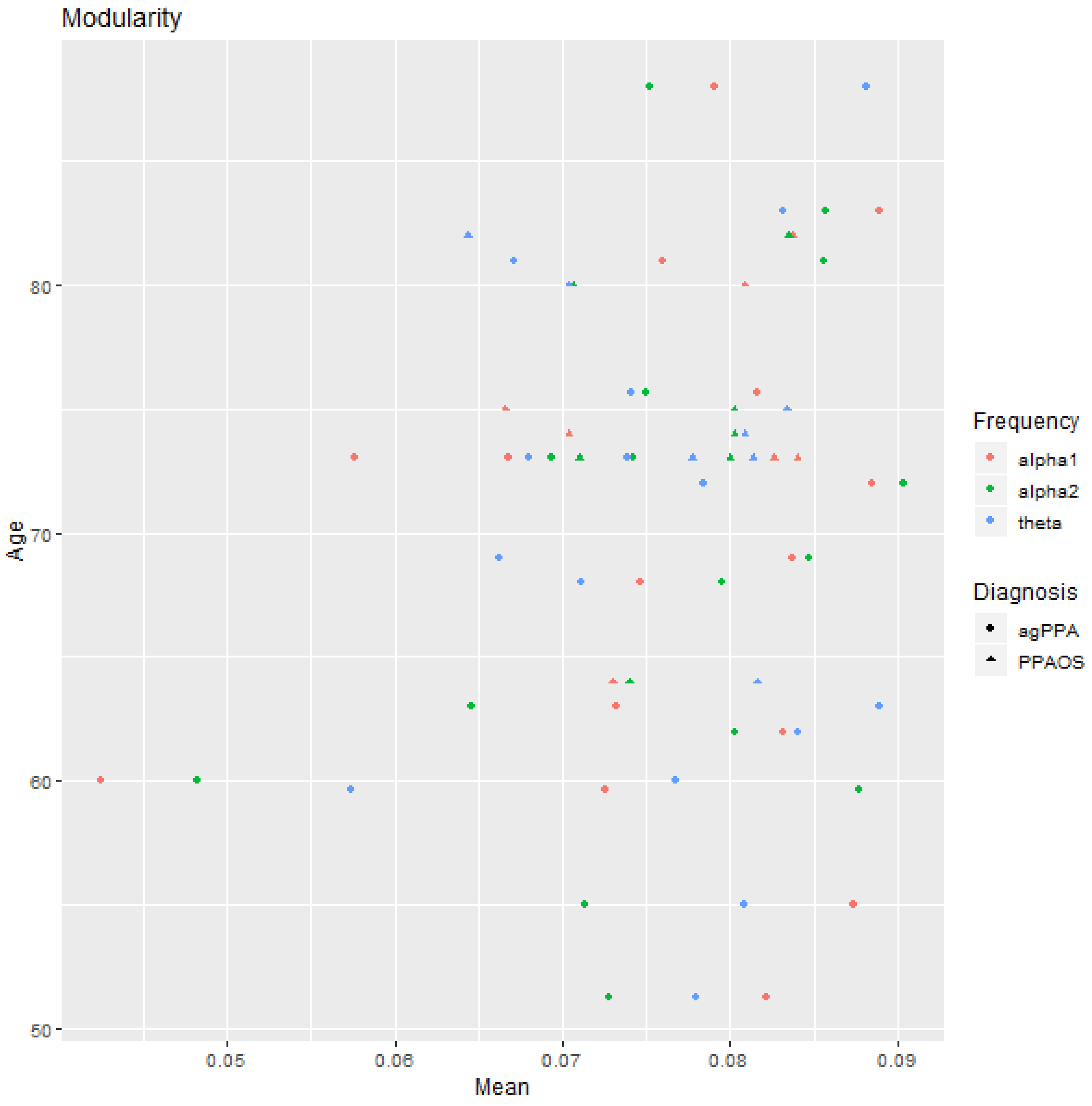
Modularity
Age
80
70
60
50
0.05
0.06
0.07
0.08
0.09
Mean
Frequency
alpha1
alpha2
theta
Diagnosis
agPPA
PPAOS

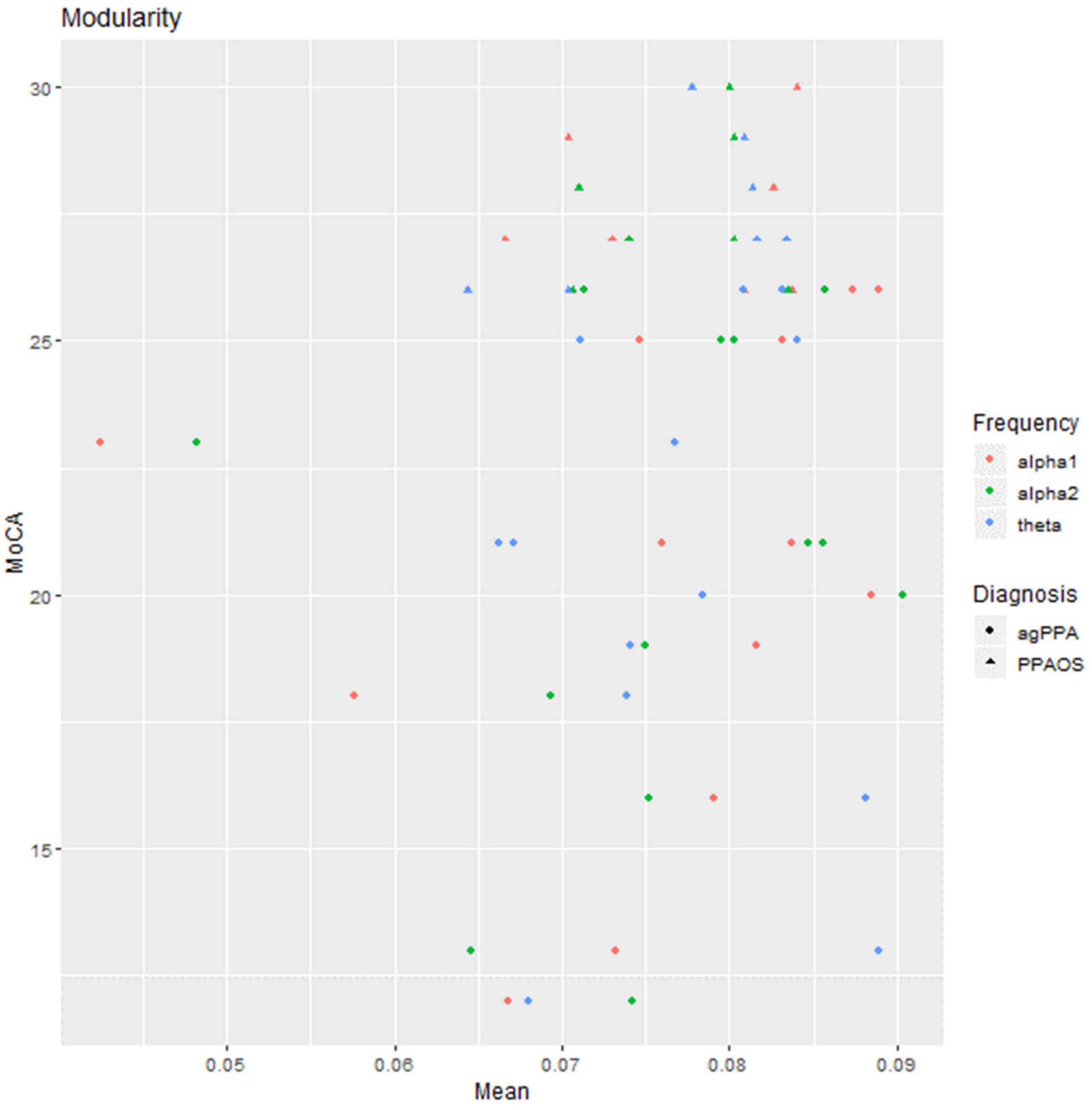

Modularity
30
25
20
15
MoCA
0.05
0.06
0.07
0.08
0.09
Mean
Frequency
alpha1
alpha2
theta
Diagnosis
agPPA
PPAOS

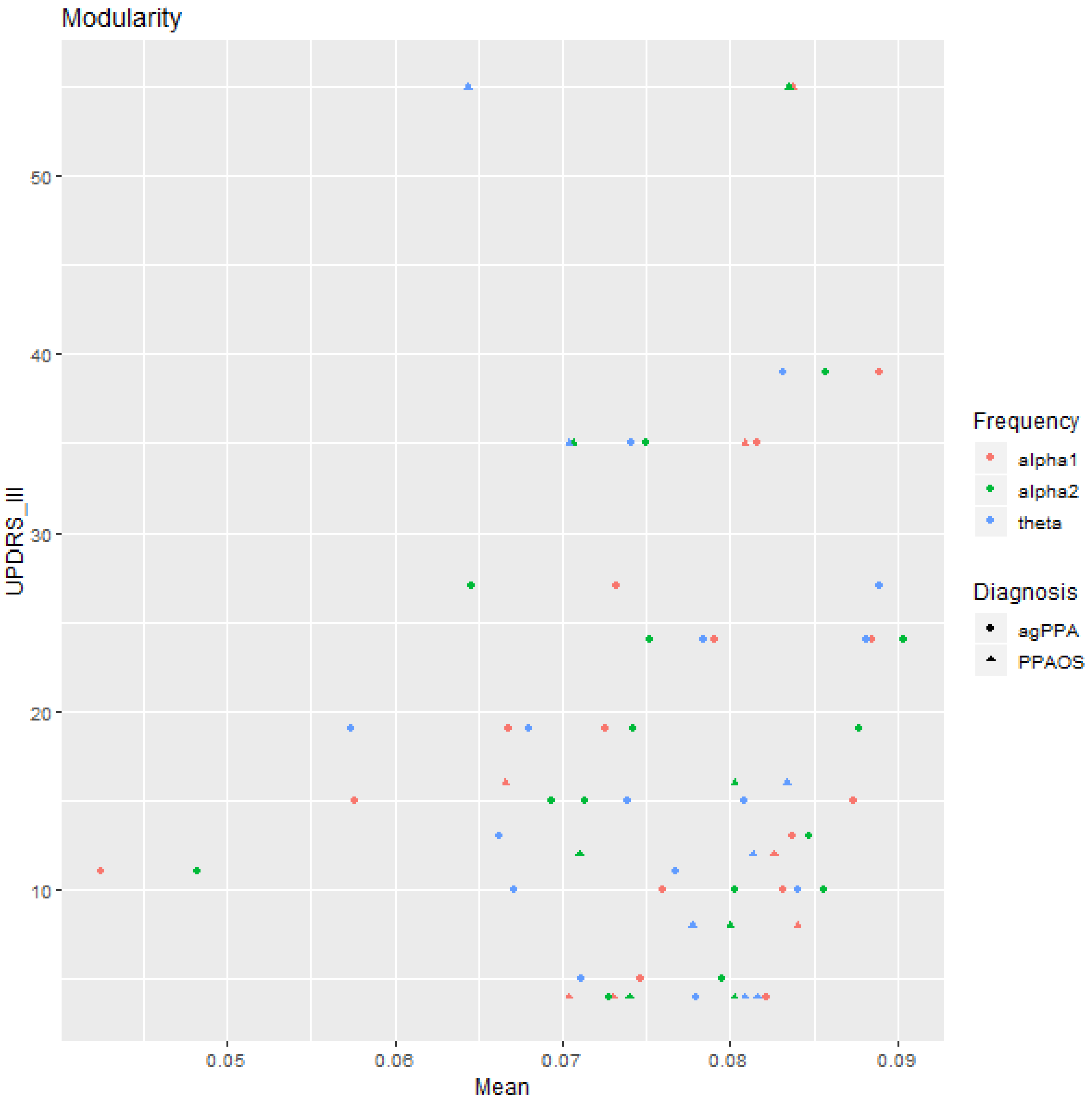

Modularity
UPDRS_III
Mean
50
40
30
20
10
0.05
0.06
0.07
0.08
0.09
Frequency
alpha1
alpha2
theta
Diagnosis
agPPA
PPAOS

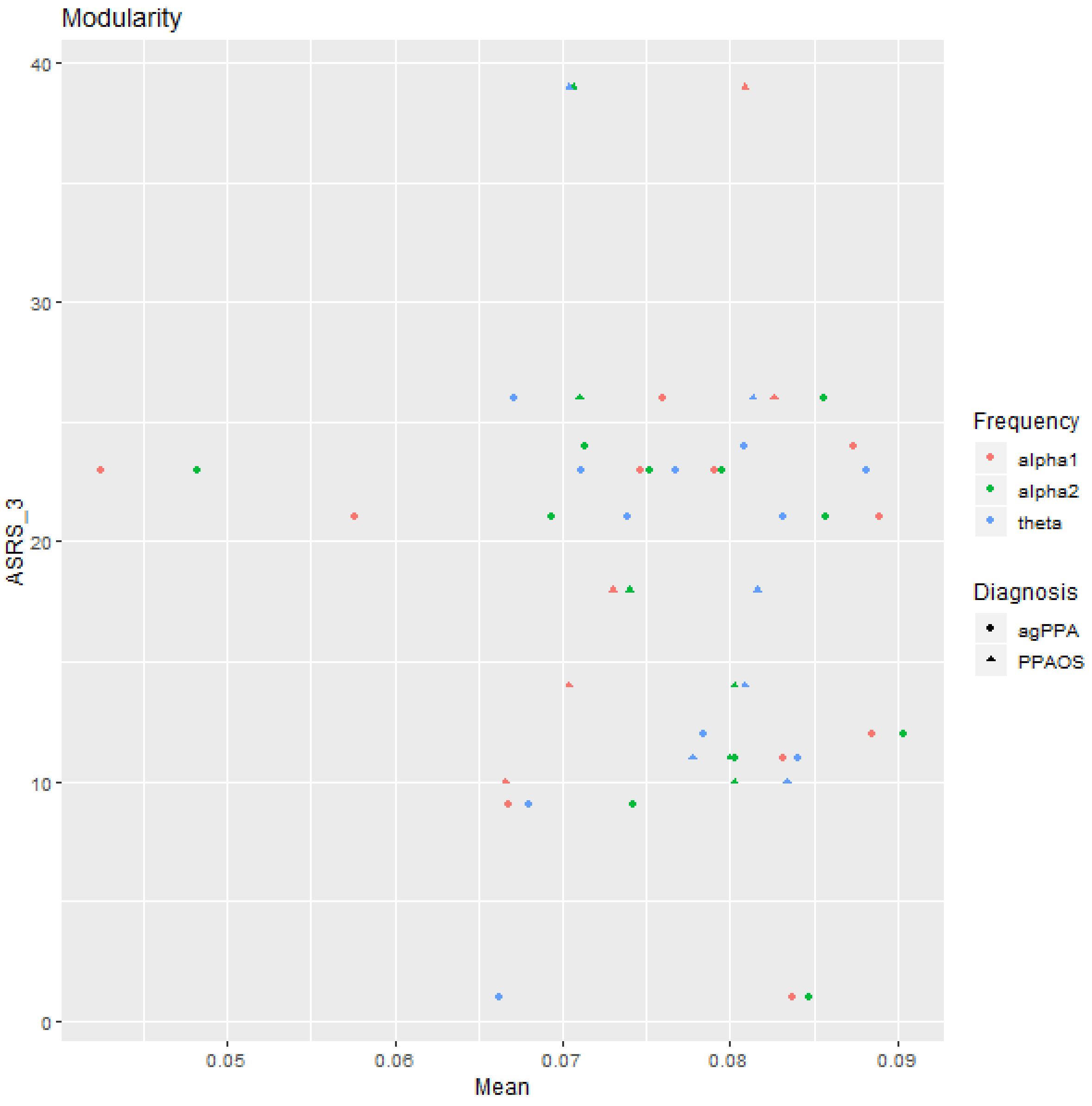

Modularity
ASRS_3
0
10
20
30
40
0.05
0.06
0.07
0.08
0.09
Mean
Frequency
alpha1
alpha2
theta
Diagnosis
agPPA
PPAOS

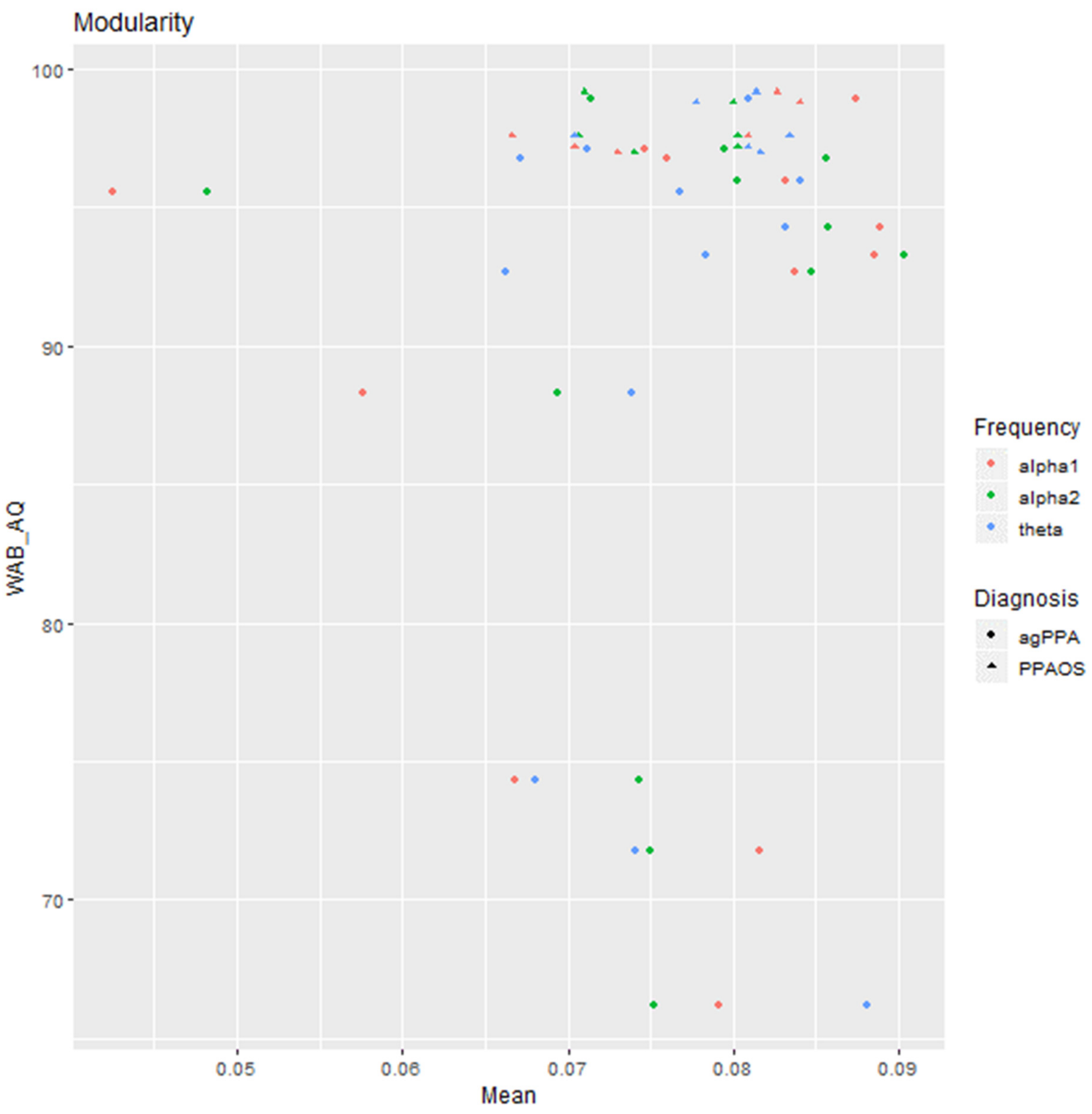

Modularity
WAB_AQ
100
90
80
70
0.05
0.06
0.07
0.08
0.09
Mean
Frequency
alpha1
alpha2
theta
Diagnosis
agPPA
PPAOS
